# Enhanced Angiogenic Potential of Electrically Stimulated Human Adipose‐Derived Mesenchymal Stem Cells (MSCs) for Ischemic Tissue Regeneration

**DOI:** 10.1002/mco2.70352

**Published:** 2025-09-09

**Authors:** Jongdarm Yi, Seungjun Lee, Chiseon Ryu, Gaeun Kim, Junghyun Kim, Jae Young Lee

**Affiliations:** ^1^ School of Materials Science and Engineering Gwangju Institute of Science and Technology (GIST) Gwangju Republic of Korea

**Keywords:** mesenchymal stem cells, electrical stimulation, paracrine effects, angiogenesis, ischemia

## Abstract

Effective treatment of ischemic disease requires the reconstruction of blood vessels through the delivery of angiogenic factors, such as chemicals, proteins, and cells. In particular, substantial efforts have focused on enhancing the therapeutic potential of mesenchymal stem cells (MSCs) for treating ischemic diseases. In this study, we investigated the use of electrical stimulation (ES) to potentiate the proangiogenic properties of human adipose‐derived MSCs. Electrically potentiated MSCs (epMSCs) were generated by applying optimized ES parameters (0.3 V, 100 Hz). EpMSCs exhibited significantly enhanced angiogenic potential, including upregulated expression of proangiogenic factors (e.g., vascular endothelial growth factor [VEGF]‐A and hepatocyte growth factor) and improved endothelial cell migration and tube formation in vitro. Transcriptomic and proteomic analyses revealed activation of key angiogenic pathways, particularly VEGFA–VEGFR2 signaling, which plays a critical role in enhancing the functionality of epMSCs. In vivo studies using a murine hindlimb ischemia model demonstrated that epMSCs enhanced blood flow recovery, induced angiogenesis, and reduced muscle atrophy more effectively than unstimulated MSCs. Overall, these findings suggest that electrical potentiation of MSCs is a promising strategy for effectively enhancing their angiogenic capabilities for treating ischemic diseases.

## Graphical abstract

This study explores the use of electrical stimulation (ES) to enhance the proangiogenic properties of mesenchymal stem cells (MSCs) for ischemic disease treatment. Electrically potentiated MSCs exhibited enhanced secretion of angiogenic factors, improved vascularization, and promoted blood flow recovery in a murine hindlimb ischemia model. These findings highlight ES as a promising strategy to advance MSC‐based therapies for ischemic tissue regeneration.

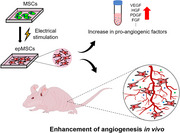

## 1 Introduction

Ischemic diseases, such as myocardial infarction, peripheral artery disease, and chronic wounds, result from inadequate blood circulation. Effective regeneration requires the formation of new blood vessels within ischemic tissues [1]. To induce angiogenesis, various therapeutic substances have been transplanted, including cells (e.g., endothelial cells) and angiogenic factors (e.g., vascular endothelial growth factor [VEGF]) [2, 3, 4]. Unfortunately, several challenges remain unaddressed, including limited cell expansion, poor survival of transplanted cells, and insufficient angiogenic stimulation [5]. Therefore, development of more effectiv and reliable therapeutic strategies is highly required.

Recently, mesenchymal stem cell (MSC)‐based therapies have attracted significant attention for treating ischemia [6]. MSCs possess unique capabilities of self‐renewal and differentiation into multiple lineages, and modulation of the microenvironment through secretion of various bioactive molecules [7]. In particular, MSCs secrete growth factors, cytokines, and extracellular vesicles, which play important roles in tissue regeneration [8, 9, 10, 11]. Their paracrine factors, such as VEGF, fibroblast growth factor (FGF), and angiopoietin‐1, can collectively induce a complex cascade in angiogenesis [12]. However, the therapeutic efficacies of these treatments remain under pathological conditions.

Accordingly, extensive studies have been performed to enhance MSC functionality by developing various approaches, including genetic modifications, stimulation (potentiation) with biochemical molecules, and the application of specialized culture conditions (e.g., 3D culture and hypoxia) [13, 14, 15, 16, 17]. More recently, biophysical cues have recently emerged as promising tools for effectively potentiating MSCs. Biophysical cues typically include mechanical, electrical, magnetic, photonic, and thermal stimulations [18]. Biophysical stimulation is cost‐effective, straightforward to implement, highly efficient, and reliable for modulating MSC functions compared with conventional biochemical stimulation [19]. Specifically, electrical stimulation (ES) with controllable parameters (e.g., voltage, frequency, and duration) can effectively influence MSC behaviors (e.g., proliferation, differentiation, and migration) by altering their transmembrane potential, receptor distribution, cytoskeleton organization, and ion influx [18, 20].

Previous studies demonstrated that ES can influence the differentiation of MSCs toward osteogenic and neurogenic lineages [21, 22, 23, 24]. Additionally, ES has been reported to influence morphology and the expression of angiogenesis‐related markers in adipose‐derived MSCs [25, 26]. However, to the best of our knowledge, comprehensive analyses of the effects of ES on MSCs’ paracrine activities, specifically with respect to angiogenesis, for ischemic disease treatment have not been conducted.

**FIGURE 1:**
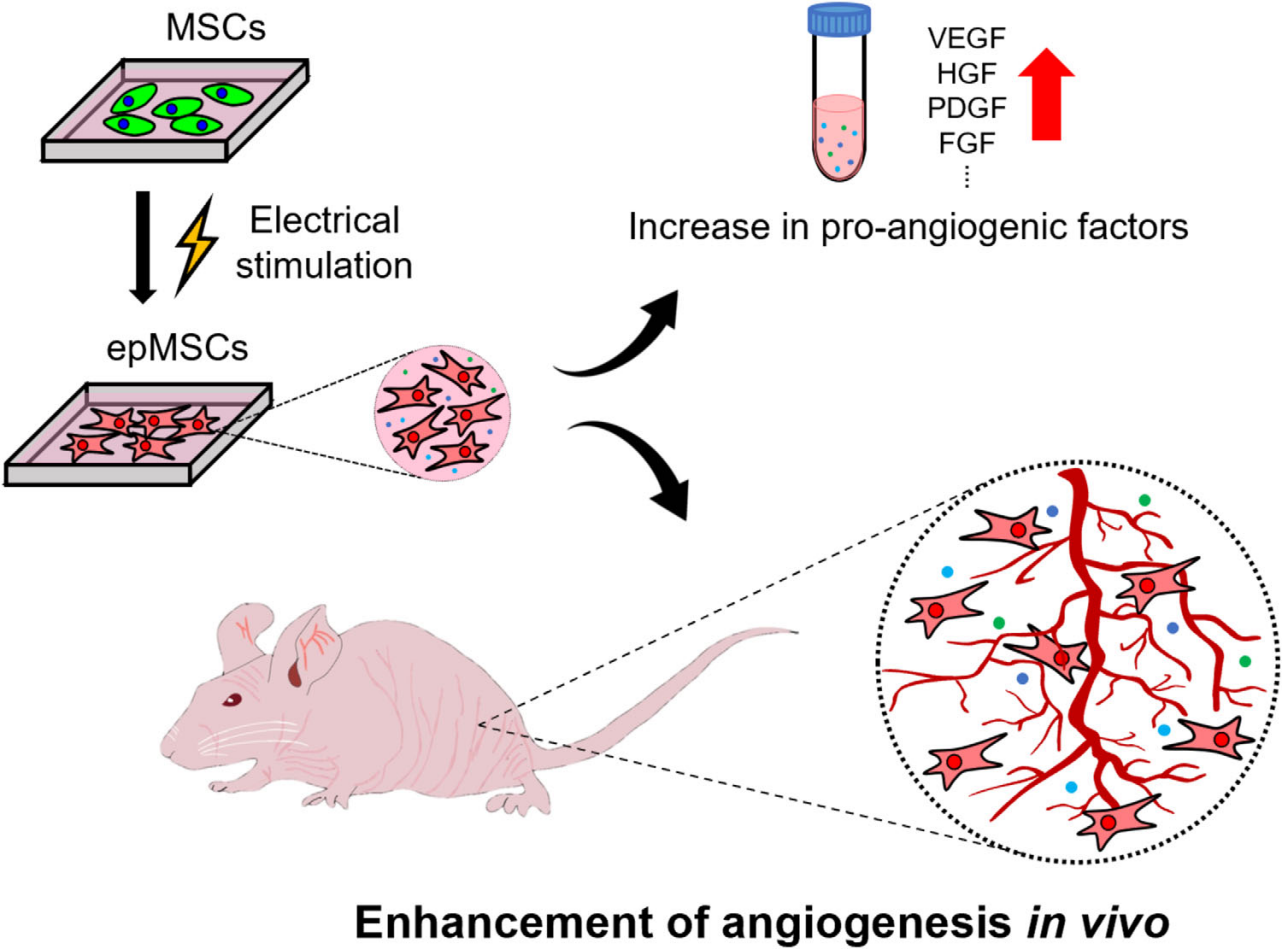
Schematic illustrations of electrically potentiated MSCs (epMSCs) and their improved angiogenic potentials.

In this study, we aimed to determine whether ES, under appropriate conditions, could enhance the angiogenic potential of MSCs and their therapeutic efficacy in the treatment of ischemic diseases. We investigated the molecular and functional changes induced in electrically potentiated MSCs (epMSCs), including proangiogenic gene/protein expression and secretion of paracrine factors. In vitro studies were conducted to assess the ability of epMSCs to promote endothelial cell migration and tube formation. Additionally, in vivo studies were performed using a murine hindlimb ischemia (HLI) model to validate their efficacy in restoring blood flow, enhancing vascularization, and mitigating muscle atrophy (Figure 1). Complementary transcriptomic and proteomic analyses were employed to elucidate the molecular mechanisms underlying ES‐induced changes in MSC function, especially regarding angiogenesis.

## 2 Results

### 2.1 Investigation of the ES Conditions

**FIGURE 2:**
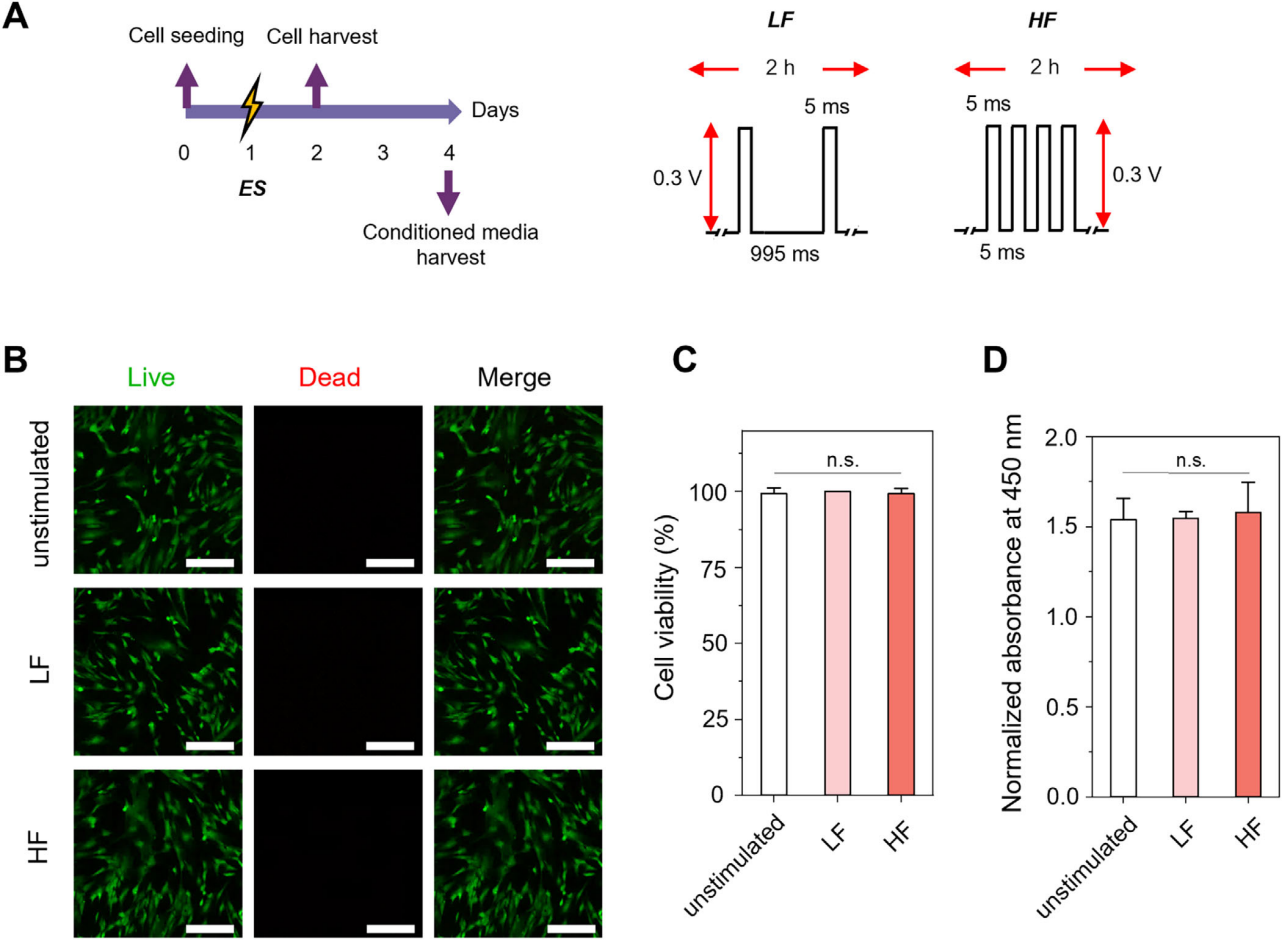
Electrical stimulation (ES) of hAD‐MSCs. (A) Schematic illustrations of in vitro experimental procedures and ES parameters. A constant voltage of 0.3 V with low frequency (LF, 1 Hz) and high frequency (HF, 100 Hz) was used for ES of hAD‐MSCs. (B) LIVE/DEAD staining images of the unstimulated MSCs (control) or MSCs stimulated with LF or HF. Green and red cells represent live and dead cells, respectively. Scale bars = 200 µm. (C) Cell viability of hAD‐MSCs (*n* = 4). Cell viability was expressed as the percentage of live cells among the total (live and dead) cells. (D) Relative metabolic activity of unstimulated or electrically stimulated MSCs. Relative metabolic activity of each group was demonstrated as absorbance at 450 nm normalized by that of the unstimulated control (*n *= 4).

MSCs were electrically stimulated under various conditions to evaluate their viability and metabolic activity. In our previous study, we identified that high voltage (0.6 V) significantly reduced MSC viability and metabolic activity, whereas 0.3 V at both 1 and 100 Hz maintained levels comparable to unstimulated controls [27]. Based on these results, we selected 0.3 V with either 1 Hz (low frequency; LF) or 100 Hz (high frequency; HF). In this study, MSCs were subjected to ES for 2 h using the laboratory‐established ES system (Figure S1) and cultured for an additional 24 h (Figure 2A). Live/dead staining revealed minimal dead cells (<3%) in all groups (Figure 2B,C). The metabolic activity of the ES group was also not significantly different from that of unstimulated MSCs (control) (Figure 2D). Furthermore, ES did not alter MSC morphology or F‐actin organization (Figure S2A). Similarly, no significant differences in cell area (Figure S2B) or aspect ratio (Figure S2C) were observed between unstimulated and stimulated MSCs. As results, the selected ES parameters (0.3 V, 1, and 100 Hz) were cytocompatible and suitable for MSC potentiation studies.

### 2.2 Expression of Angiogenic Markers in epMSCs

**FIGURE 3:**
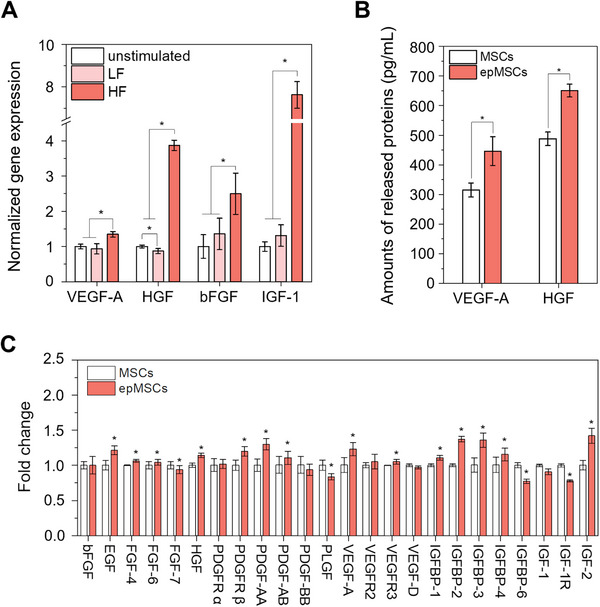
Expression of typical angiogenic markers in epMSCs. (A) Relative mRNA expression of VEGF‐A, HGF, bFGF, and IGF‐1 genes. Expression of each gene was normalized to that of GAPDH (*n* = 4). (B) Quantification of VEGF‐A and HGF produced from MSCs and epMSCs (*n* = 4). (C) Fold change in the mean pixel density of each growth factor produced by MSCs and epMSCs (*n* = 4). CMs from MSCs and epMSCs were analyzed using a human growth factor antibody array to detect growth factor production in MSCs. Gene expression was normalized to GAPDH using the 2^−ΔΔCt^ method. An asterisk (*) denotes a statistically significant difference (*p* < 0.05).

Potential alterations in the angiogenic potential of MSCs induced by ES were investigated by assessing the mRNA expression of key angiogenic factors (i.e., VEGF‐A, hepatocyte growth factor [HGF], basic FGF [bFGF] and insulin‐like growth factor 1 [IGF‐1]) [28] (Figure 3A). Their gene expression levels were significantly higher in the HF group than those in the unstimulated and LF groups. Specifically, ES with HF led to 1.3‐, 3.9‐, 2.5‐, and 7.6‐fold increases in VEGF‐A, HGF, bFGF, and IGF‐1 expression, respectively, compared with unstimulated controls. In contrast, the LF group showed no significant changes in VEGF‐A, bFGF, and IGF‐1, and downregulated HGF expression. Altogether, HF (0.3 V, 100 Hz) was determined as the appropriate ES condition for subsequent studies. MSCs secrete a diverse range of therapeutically active molecules, including growth factors, cytokines, microRNAs, and other small molecules [29]. Hence, we quantified the secretion of representative proangiogenic proteins (i.e., VEGF and HGF) in the conditioned media (CM). The epMSCs produced greater amounts of VEGF (1.5‐fold) and HGF (1.3‐fold) compared with unstimulated MSCs (Figure 3B). Time‐course profiling revealed that this increase was rapid within 12 h and sustained for up to 72 h (Figure S3A,B). Staining of human growth factor arrays revealed that the production of various secretomes from MSCs were significantly altered by ES, including epidermal growth factor (EGF), FGF, HGF, platelet‐derived growth factor (PDGF), PDGF receptor, VEGF, IGF, and IGF‐binding protein (Figures 3C and S4). VEGF‐A and HGF levels were significantly higher in epMSCs than MSCs, which was consistent with the quantitative real‐time PCR (qRT‐PCR) and enzyme‐linked immunosorbent assay (ELISA) results (Figure 3A,B). Altogether, our results indicate that ES significantly enhances the angiogenic potential of MSCs by upregulating key factors, such as VEGF and HGF.

### 2.3 In Vitro HUVEC Culture for Evaluation of Angiogenic Functions of epMSCs

**FIGURE 4:**
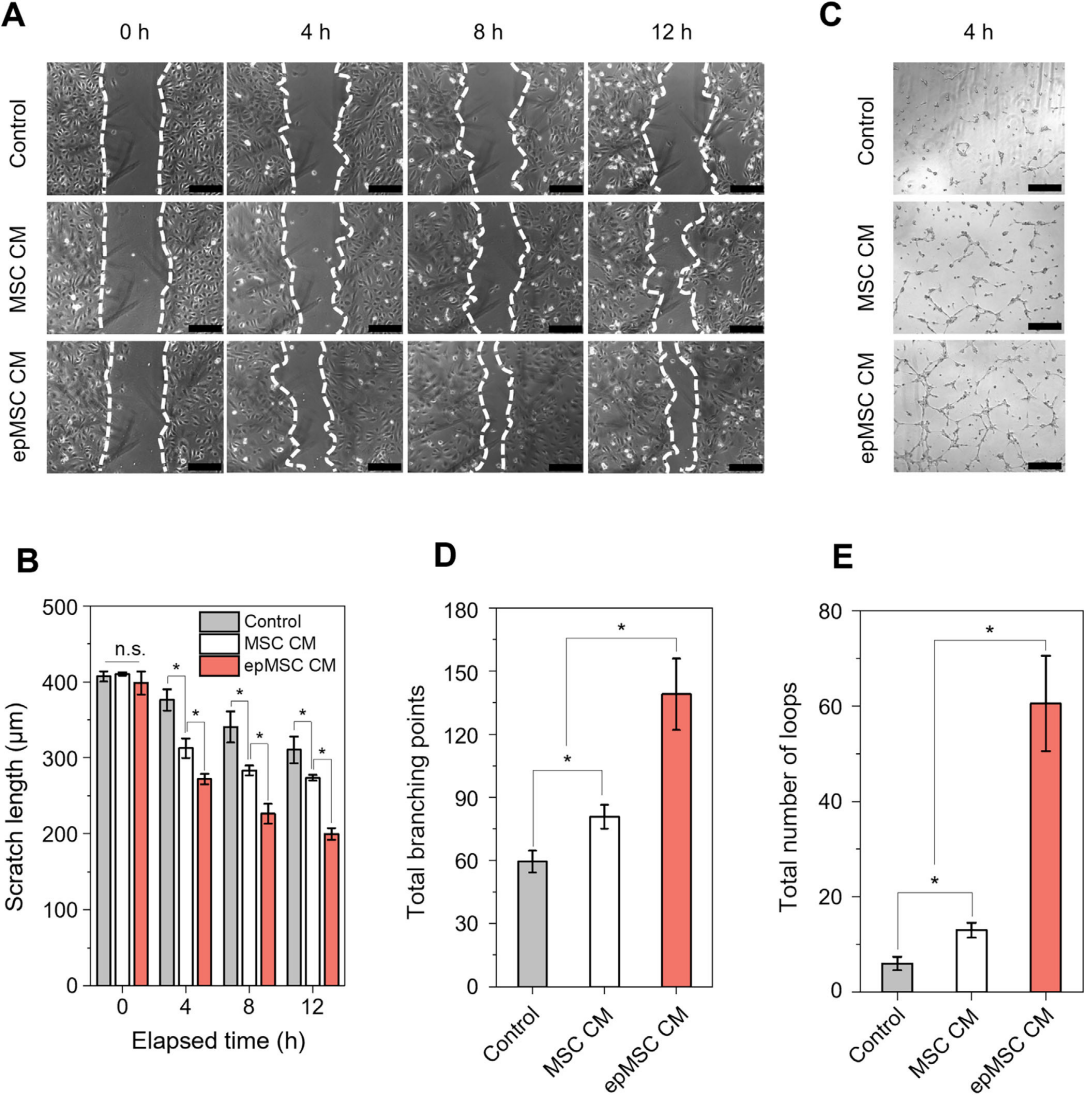
Functional assays of the angiogenic potentials of epMSCs with in vitro HUVEC culture with conditioned medium (CM). HUVECs were cultured with 1:1 mixed serum‐free endothelial cell medium (ECM) with MEM‐α, MSC CM, and epMSC CM, respectively. (A) Representative optical micrographs of HUVEC scratches in individual groups at 0, 4, 8, and 12 h. Scale bar = 200 µm. (B) The corresponding scratch lengths (*n* = 4). (C) Representative optical micrographs of HUVECs tube formation in individual groups at 4 h. Scale bar = 200 µm. (D) The corresponding total branching points (*n* = 4) and (E) total number of loops of tubes at 4 h (*n* = 4). An asterisk (*) denotes a statistically significant difference (*p* < 0.05).

To evaluate the proangiogenic effects of epMSCs, in vitro scratch closure and tube formation assays were performed by culturing HUVECs with CM from MSCs or epMSCs. The migration of HUVECs was quantitatively assessed using the scratch closure assay at 4‐h intervals (Figure 4A). HUVECs cultured with CM mixtures of MSCs or epMSCs showed significantly faster scratch closure after 4 h of incubation compared with the control; scratch closure in the epMSC CM group was superior to that in the MSC CM group. For instance, the epMSC CM group exhibited a 49.7 ± 3.4% reduction in scratch at 12 h compared with the initial scratch, whereas the control and MSC CM groups demonstrated scratch reductions of 23.8 ± 3.2 and 33.3 ± 1.1%, respectively (Figure 4B). Moreover, culture with the CM of MSCs (MSC CM and epMSC CM) significantly increased the tube formation of HUVECs compared with that with the serum‐free control group (Figure 4C). The epMSC CM group induced significantly higher numbers of branching points and closed loops compared with the other groups (Figure 4D,E). Overall, the results indicated that epMSCs appeared to secrete more proangiogenic molecules that could enhance the migration and tubulogenesis of HUVECs.

### 2.4 In Vivo Plug Assay

In vivo angiogenic activity of the epMSCs was examined using a Matrigel‐based plug assay. Matrigel mixed with PBS (control), MSCs, or epMSCs was subcutaneously injected into mice, and the plugs were harvested on day 7 (Figure S5). Plugs from the control group were transparent with no visible blood vessels, whereas those from the MSC group sparsely formed blood vessels. In contrast, the plugs from the epMSC group exhibited a reddish color with noticeable blood vessels (Figure S5A). Hematoxylin and eosin (H&E) staining and von Willebrand factor (vWF) immunostaining revealed that the plugs of the control, MSC, and epMSC groups showed no vessels, a few blood vessels, and dense blood vessels, respectively. The hemoglobin contents were significantly higher in the MSC groups, especially in the epMSC group (Figure S5B). Additionally, the vWF‐positive area in the epMSC group was 2.1‐fold larger than that in the MSC group (Figure S5C). Macroscopic observations and histological analyses consistently demonstrated that epMSCs significantly promote blood vessel formation in vivo.

### 2.5 Transcriptomic and Proteomic Analyses of epMSCs

**FIGURE 5:**
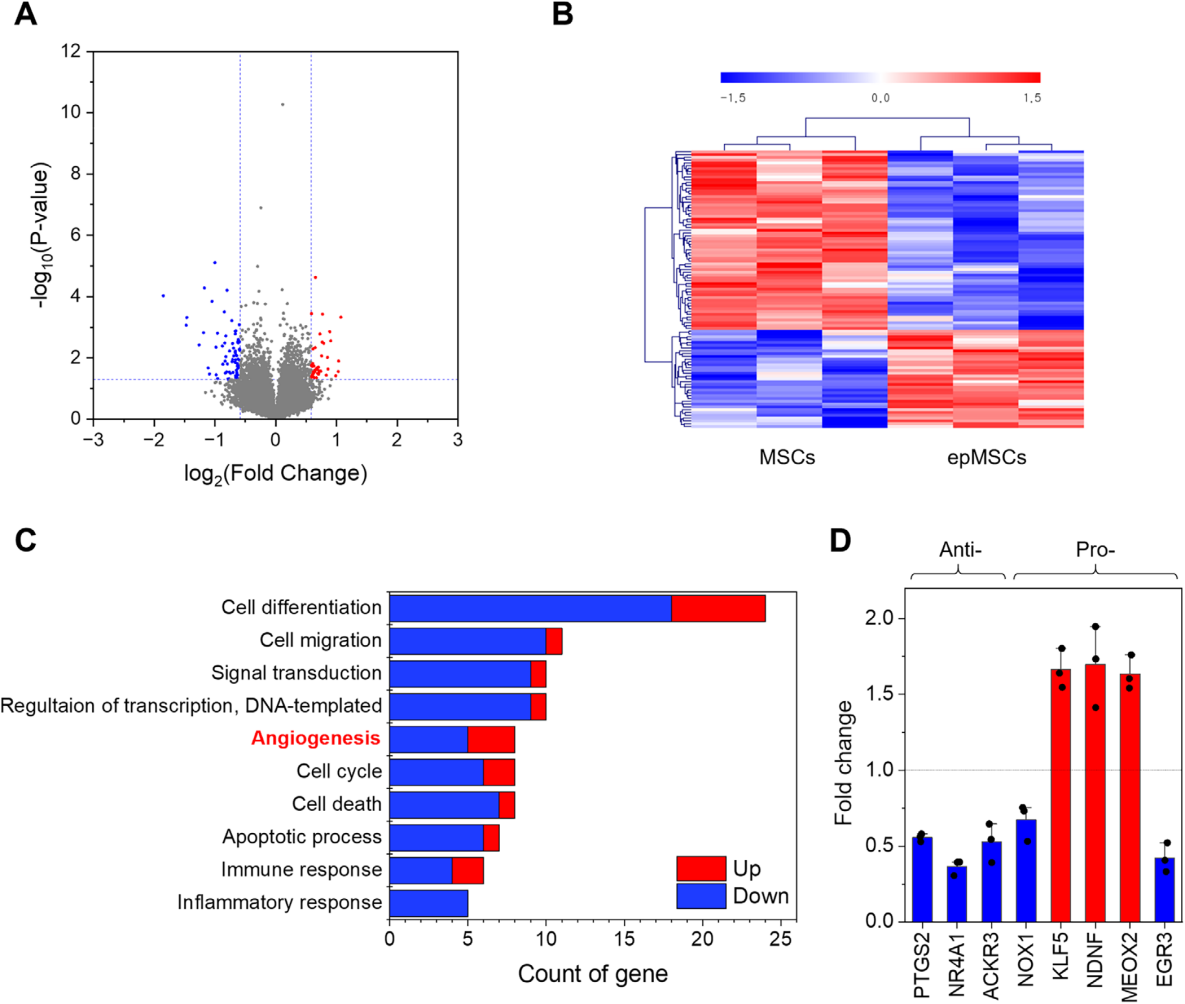
Quantitative RNA sequencing analysis. (A) Volcano plot of differentially expressed genes (DEGs). The red and blue dots indicate the upregulated and downregulated genes in the epMSCs compared with those in the MSCs, respectively. (B) Hierarchical clustering analysis of DEGs between the MSCs and epSCs with a color scale indicating upregulation (red) and downregulation (blue). (*n *= 3 per group). (C) Ten major gene ontology (GO) analysis of the genes of which expressions were significantly influenced by ES. (D) Gene expression profile associated with GO terms related to angiogenesis. Genes depicted in blue and red indicate upregulation and downregulation of angiogenesis, respectively.

Comprehensive transcriptomic and proteomic analyses were performed to elucidate the molecular mechanisms underlying the enhanced angiogenic potential of epMSCs. Quantitative RNA sequencing revealed differential expressions of 23,587 genes between MSCs and epMSCs (Figure 5A). Notably, 99 genes in the epMSC group exhibited 1.5‐fold or higher changes (*p *< 0.05) in expression compared with those in the MSCs group, in which 35 genes were upregulated and 64 genes were downregulated (Figure 5B). Gene ontology (GO) analysis identified 10 major ontologies involving 48 genes affected by ES. These genes were linked to diverse biological processes, such as cell differentiation, migration, angiogenesis, apoptosis, cell cycle, and various signal transduction pathways (Figure 5C). In particular, eight genes were associated with angiogenesis (Figure 5D). Among them, three genes (PTGS2, NR4A1, and ACKR3) inhibit angiogenesis, and five genes (NOX1, KLF5, NDNF, MEOX2, and EGR3) promote angiogenesis [30, 31, 32, 33, 34, 35, 36]. Although NOX1 and EGR3 expressions decreased, overall gene expression profile suggested a proangiogenetic tendency. The interactions among proteins corresponding to differentially expressed genes (DEGs) are demonstrated in Figure S6. Analysis of functional enrichment and clustering revealed that blood vessel development (GO:0001568), with proteins highlighted in red, was the most prominently enriched among the identified biological processes within the network. These results suggest that ES induces MSC gene expression associated with angiogenesis.

**FIGURE 6:**
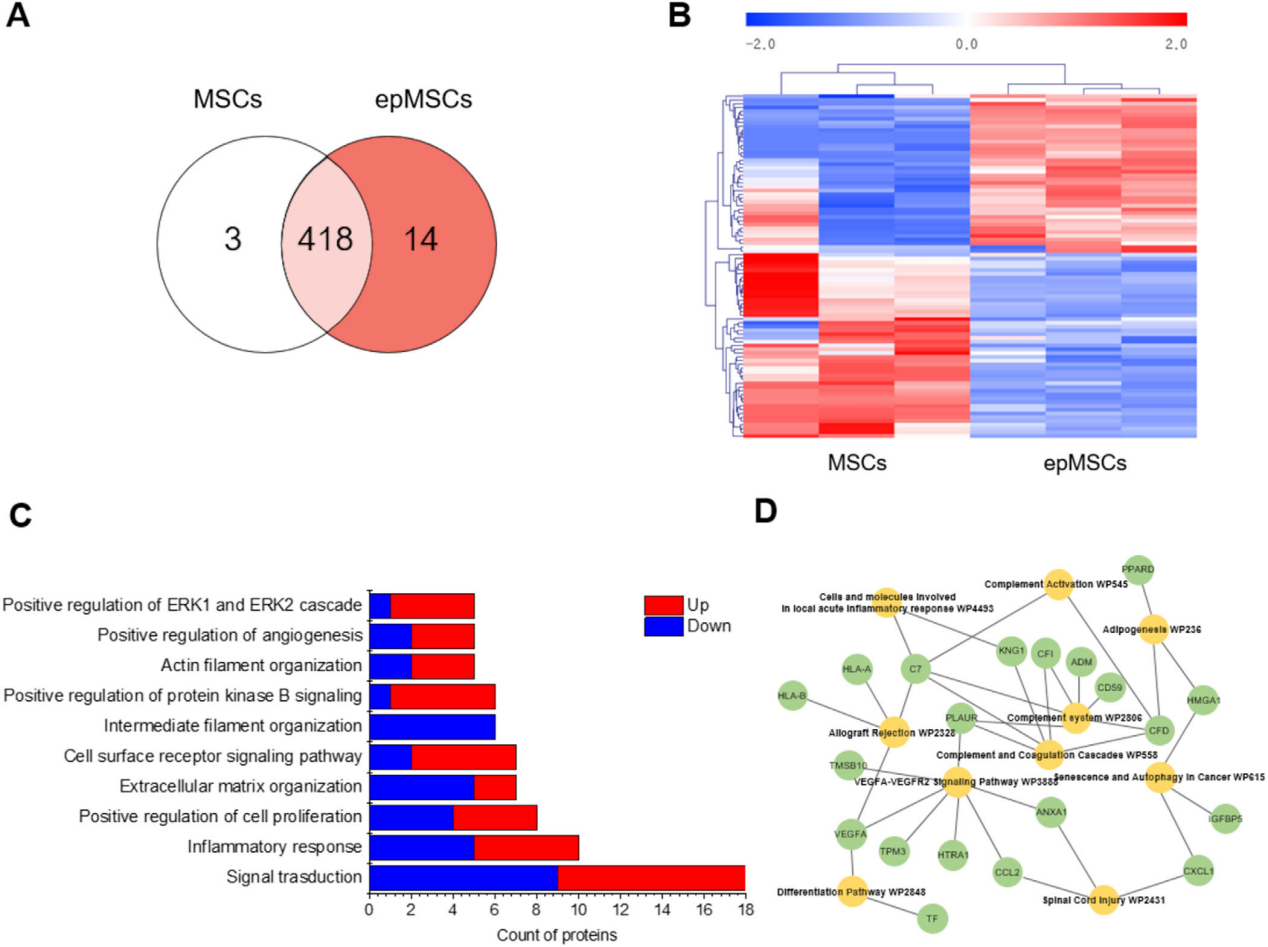
Proteomics analysis of MSCs and epMSCs. (A) Venn diagram illustrating the distribution of proteins identified the CM of MSCs and epMSCs. (B) Hierarchical clustering analysis of the differentially expressed proteins between MSC CM and epMSC CM with a color scale indicating downregulation (blue) and upregulation (red). (C) GO analysis highlighting the top 10 biological processes significantly altered by ES with. (D) Functional enrichment analysis of differentially expressed proteins with the top 10 significantly enriched GO terms related to signaling pathways.

Proteomics analysis was performed to quantitatively assess the proteins secreted by MSCs and epMSCs. A total of 435 proteins were identified, including 418 proteins identified in both groups. Moreover, 14 and 8 proteins were identified exclusively in the epMSC and MSC groups, respectively (Figure 6A). With a fold‐change (>1.5), 42 proteins showed high abundance, and 49 proteins showed low abundance in the epMSC group (Figure 6B). Proteins with high or low abundance in epMSCs were further categorized based on GO (Figure 6C). GO analysis of the differentially expressed proteins indicated the upregulation of proteins involved in several important biological processes, including positive regulation of cell proliferation, extracellular matrix organization, angiogenesis, ERK1/2 signaling, and protein kinase B (Akt) signaling, and cell surface receptor signaling. Pathway enrichment analysis further revealed significant connections between the epMSC secretome and key proangiogenic pathways [37]. The top 10 enriched pathways included the complement system, complement and coagulation cascades, and the VEGFA–VEGFR2 signaling pathway (Figure 6D). Moreover, several proangiogenic (e.g., PLAUR) proteins and immune‐modulating proteins (e.g., CCL2 (MCP‐1) and Annexin A1 (ANXA1)) were identified in the epMSC secretome. Collectively, these findings demonstrate that ES enhances the angiogenic potential of MSCs by influencing multiple interconnected pathways for sustained vascular growth.

### 2.6 Assessment of Therapeutic Efficacy of epMSCs in a HLI Model

**FIGURE 7:**
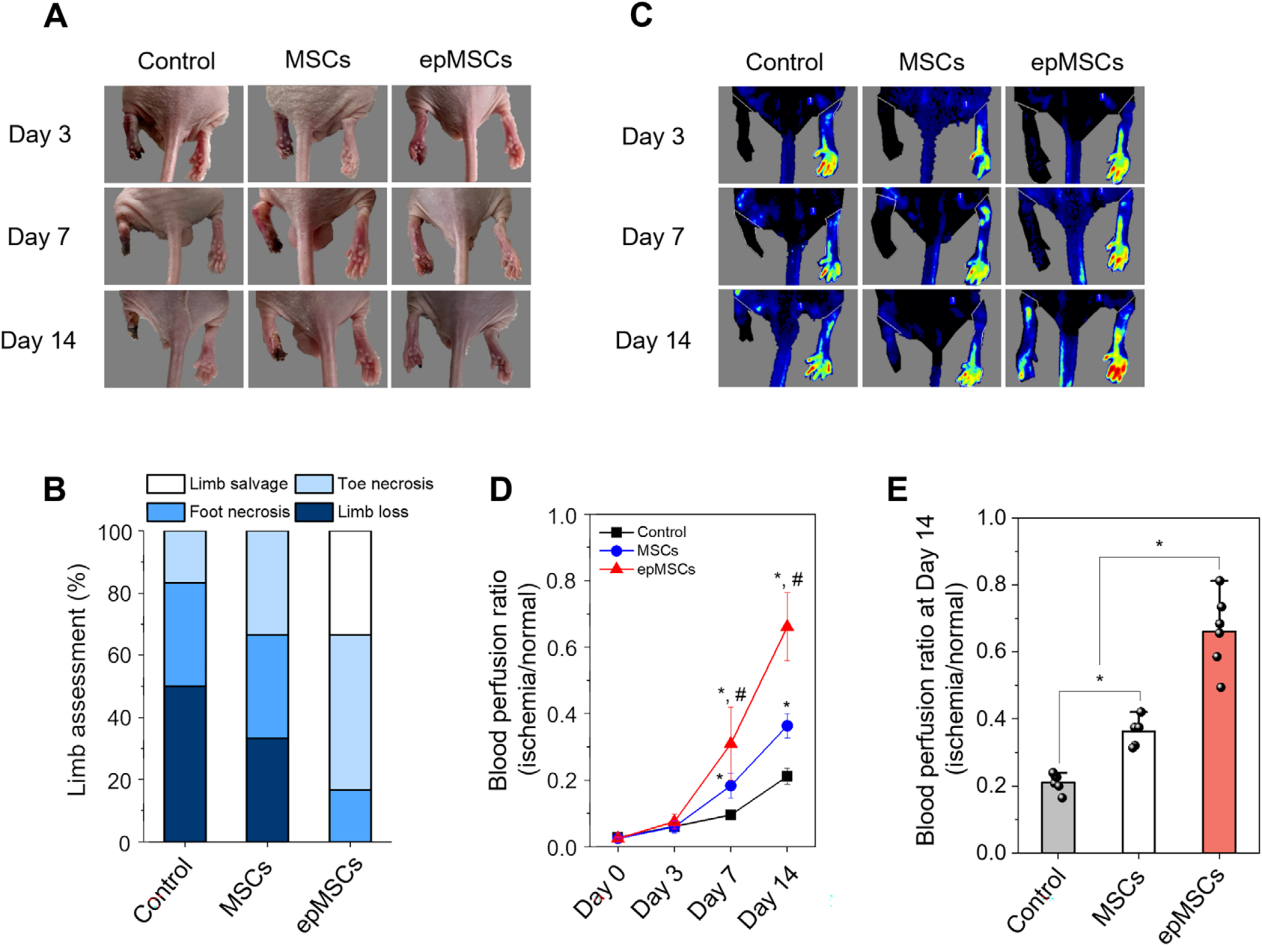
Assessment of the therapeutic activity of epMSCs in a mouse hindlimb ischemic model. (A) Representative photographs of ischemic hindlimbs on days 3, 7, and 14 after surgery and treatment. (B) Limb assessment on day 14. (C) Laser Doppler perfusion imaging of the ischemic hindlimbs on days 3, 7, and 14. (D) Blood perfusion ratio of the ischemic limb in each group (*n* = 6). An asterisk (*) and a crosshatch (#) denote statistically significant difference (*p* < 0.05) when compared with the control and MSC groups, respectively. (E) Blood perfusion ratios of each group on day 14 (*n* = 6). An asterisk (*) denotes a statistically significant difference (*p* < 0.05).

The therapeutic efficacy of epMSCs was examined using a mouse HLI model. The experimental groups included PBS (control), unstimulated MSCs, and epMSCs. Each sample was injected intramuscularly into ischemic limbs (Figure S7). The extent of limb salvage was monitored in the feet of HLI‐induced mice and quantified on day 14 (Figures 7A,B and S8). By day 3, blackened paws were observed in all experimental groups, which progressed to toe loss by day 7 in the control and MSC groups. By day 14, foot and toe necrosis were prevalent in the control and MSC groups. In contrast, the epMSC group exhibited minimal necrosis and no limb loss. Laser Doppler perfusion imaging was performed to monitor blood flow over a 2‐week period after the operation (Figure 7C). On day 3, blood perfusion was minimal in all groups (Figure 7D). However, on days 7 and 14, blood perfusion ratios gradually recovered in all groups. The epMSC group consistently exhibited the highest blood perfusion ratio among all groups. On day 14, blood flow ratios were 0.21 ± 0.03 (control), 0.36 ± 0.04 (MSC), and 0.67 ± 0.11 (epMSC) (Figure 7E). These results suggest that epMSCs effectively promote angiogenesis, preventing limb necrosis and recovering blood flow in HLI.

**FIGURE 8:**
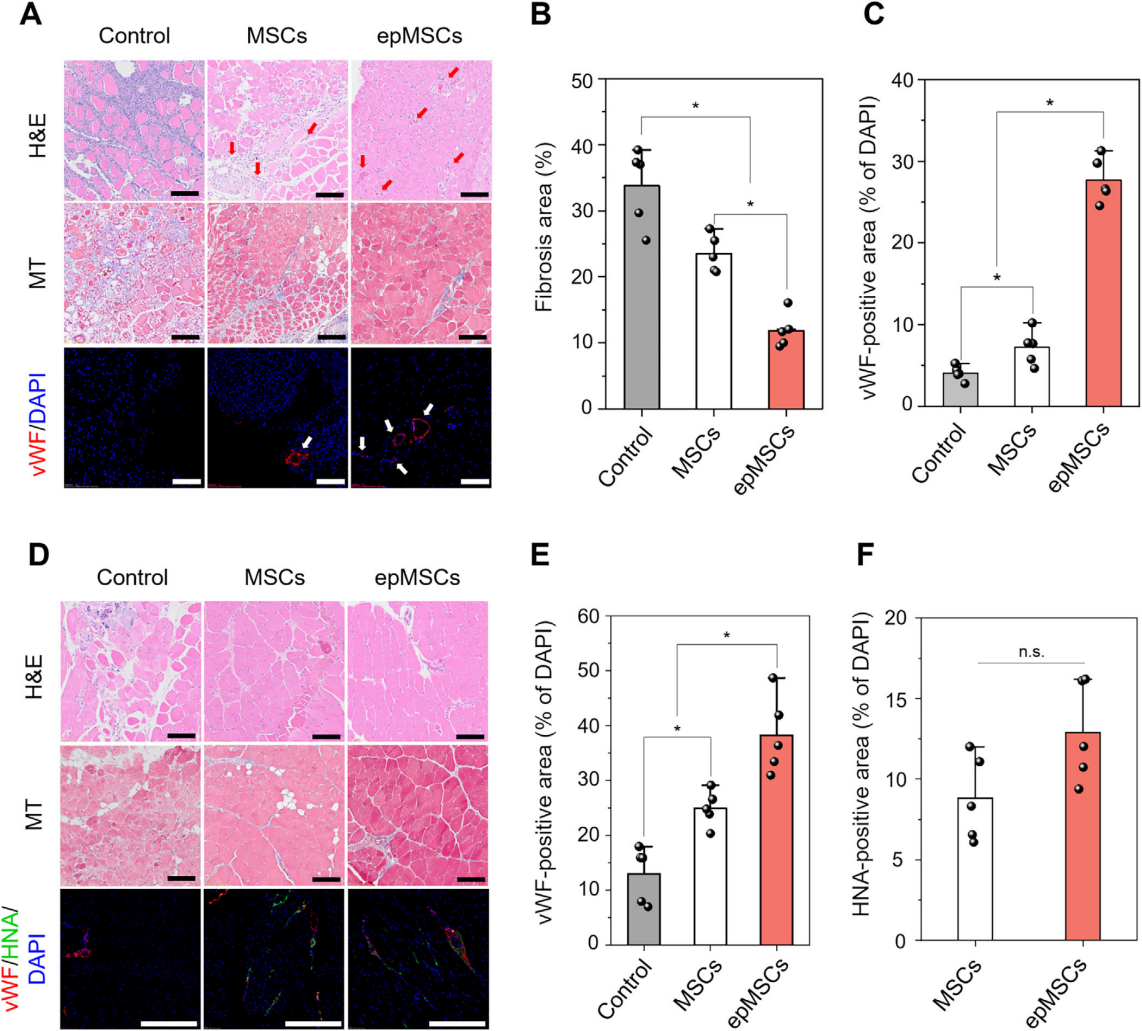
Histological analysis of the gastrocnemius and quadriceps muscles harvested on day 14 after surgery and treatment. (A) Representative images of H&E, Masson's trichrome (MT), and vWF staining of gastrocnemius in each group. Red and white arrows indicate blood vessels. (B) Fibrosis (*n* = 5) and (C) vWF‐positive areas (*n* = 5). vWF‐positive area was normalized to the DAPI‐positive area in each group. (D) Representative images of H&E, MT, vWF, and HNA staining of quadriceps in each group. (E) vWF‐positive (*n* = 5) and (F) HNA‐positive areas (*n* = 5). vWF‐positive or HNA‐positive areas were normalized to the DAPI‐positive areas in each group. Scale bars = 100 µm. An asterisk (*) denotes a statistically significant difference (*p* < 0.05).

Ischemic injury typically causes the apoptosis of skeletal muscle fibers and structural damage [38]. In our study, because MSCs can enhance blood circulation and facilitate improved limb salvage by secreting various angiogenic growth factors and immunomodulatory substances in the ischemic limb, we expected that transplantation of MSCs or epMSCs would alleviate muscle atrophy [32]. Notable infiltration of inflammatory cells and structural disorganization were observed in gastrocnemius tissues from the control group. Conversely, the MSC group displayed lower cell infiltration and moderate vascularization. The epMSC group exhibited structural integrity, blood vessels, and centrally located nuclei within the muscle fibers (Figure 8A), which are widely recognized indicators of appropriate muscle regeneration [39].

Fibrosis was significantly reduced in the MSC and especially epMSC groups. For example, the fibrosis areas of the control, MSC, and epMSC groups were 33.8 ± 5.9, 23.5 ± 2.8, and 11.9 ± 2.6, respectively (Figure 8B). In addition, Immunostaining for vWF confirmed increased vascularization in epMSC‐treated muscles (Figure 8C). The vWF‐positive area in the epMSC group was 6.8‐ and 3.8‐fold larger than that in the control and MSC groups, respectively. Similarly, the quadriceps muscles of the MSC‐treated group displayed improved structural integrity, reduced cell infiltration, and increased blood vessel formation (Figure 8D). Interestingly, fat deposition, typically caused by metabolic and musculoskeletal damages [40], was observed in the control and MSC groups, but markedly reduced in the epMSC group. Additionally, larger areas of vWF‐positive cells were found in the MSC (25.0 ± 3.2%) and epMSC (38.3 ± 7.1%) groups than in control group (13.0 ± 5.1%) (Figure 8E). Human nuclear antigen (HNA) immunostaining revealed the enhanced viability and residence of the transplanted MSCs (Figure 8F). HNA‐positive areas were 8.8 ± 2.4 and 12.9 ± 2.8% for the MSC and epMSC groups, respectively. Costaining for HNA and vWF revealed limited colocalization (Figure S9). The percentages of vWF‐positive area within the HNA‐positive population were 12.2 ± 2.2 and 13.2 ± 2.5% in the MSC and epMSC groups, respectively, in which the difference was not significant. These results suggest that most transplanted cells contributed to vascularization through paracrine mechanisms rather than direct endothelial differentiation.

## 3 Discussion

Although ES can modulate cell behavior, inappropriate ES parameter can generate harmful by‐products, including hydrogen peroxide, hydroxyl ions, and other intermediates, potentially leading to cellular damage [41]. However, our in vitro studies confirmed the optimized ES conditions (0.3 V, 100 Hz for 2 h) did not impair cell viability, morphology, or metabolic activity.

The major finding of this study is that ES significantly increases the expression and secretion of key proangiogenic factors, including VEGF‐A, HGF, bFGF, and IGF‐1. Among these, VEGF‐A and HGF are well‐established regulators of vascular growth and remodeling. The upregulation of these angiogenic genes and proteins indicates that ES rapidly activates intracellular signaling pathways and sustains the production of angiogenic factors. Notably, the enhancement was frequency dependent. HF stimulation induced greater gene and protein expression, which may be attributed to more efficient intracellular penetration and transcriptional activation [42].

Functional assays further confirmed that CM from epMSCs significantly enhanced migration and tubulogenesis of HUVECs. These in vitro results are in a good agreement with the in vivo Matrigel plug results, where epMSCs significantly increased hemoglobin content and vessel density. In the HLI model, epMSC treatment improved blood perfusion, reduced tissue necrosis, and attenuated muscle fibrosis. The observed histological improvements indicate the proangiogenic and tissue‐protective effects of epMSCs. Moreover, limited colocalization of HNA and vWF in transplanted tissues suggests that epMSC‐mediated therapeutic effects are predominantly paracrine rather than through direct differentiation.

To elucidate the molecular mechanisms underlying these enhanced effects, transcriptomic and proteomic analyses were performed. RNA sequencing identified differentially expressed genes associated with angiogenesis, apoptosis, and immune responses. Especially, proangiogenic genes such as KLF5 and NDNF were upregulated, while angiogenesis‐inhibitory genes were downregulated. Proteomic analysis supported these findings, highlighting increased expression of proteins related to the VEGFA–VEGFR2, PI3K/Akt, and ERK1/2 pathways, which play important roles in endothelial cell functions [43, 44]. In addition, secretome profiling identified the upregulation of PLAUR, CCL2, and ANXA1, which are known to mediate extracellular matrix remodeling, immune modulation, and endothelial stabilization, respectively. These overall trends in proangiogenic transcriptional and proteomic profiles can explain the enhanced angiogenic potential of epMSCs observed both in vitro and in vivo.

Importantly, ES significantly enhanced the regenerative potential of MSCs by promoting their angiogenic capacity and reducing tissue damage in various ischemic diseases, such as peripheral artery disease or myocardial infarction. This approach offers a cost‐effective, scalable strategy that can be integrated into current MSC manufacturing processes. The ES parameters used in this study (low‐voltage: 0.3 V; high‐frequency: 100 Hz) are safe and clinically feasible. While we demonstrated therapeutic efficacy 14 d postsurgery and treatment in this study, the long‐term studies will be necessary to assess the sustainability of these improvements. For clinical translation of epMSC, several issues should be addressed, including the establishment standardized ES protocols and production conditions, long‐term safety and efficacy, and validation in large animal models.

## 4 Conclusion

This study demonstrated that ES significantly enhances the angiogenic potential of MSCs, presenting a promising strategy for MSC‐based therapies for ischemic tissue regeneration. EpMSCs exhibited upregulation of key angiogenic factors, such as VEGF‐A and HGF, leading to enhanced endothelial cell migration and tube formation in vitro. Comprehensive transcriptomic and proteomic analyses further revealed that ES substantially activates angiogenic signaling pathways, including the VEGFA–VEGFR2 signaling. In vivo studies revealed that epMSC transplantation substantially promoted angiogenesis, enhanced blood flow recovery, and reduced muscle atrophy in ischemic tissues compared with unstimulated MSCs. We demonstrated the therapeutic efficacy of epMSCs and the mechanisms underlying their enhanced angiogenic capabilities. Altogether, this study establishes ES as a powerful approach to enhance the regenerative properties of MSCs, thereby advancing their therapeutic potential for treating ischemic diseases. Future investigations are necessary to optimize the ES parameters and elucidate the underlying mechanisms to fully harness the benefits of epMSCs in clinical applications.

## 5 Materials and Methods

### 5.1 Cell Culture

Human adipose‐derived MSCs (hAD‐MSCs) (PromoCell, Heidelberg, Germany) were maintained in MEM‐α (Gibco, Grand Island, NY, USA) supplemented with 10% fetal bovine serum (FBS; Gibco) and 1% antibiotic–antimycotic (100×; Gibco) in an incubator with 5% CO_2_ at 37°C used at passage number 6. HUVECs (Gibco) were cultured in endothelial culture medium (ECM; SceinCell, CA, USA) supplemented with 5% FBS, 1% penicillin–streptomycin, and 1% endothelial cell growth supplement in an incubator with 5% CO_2_ at 37°C and used at passage number 6.

### 5.2 Electrical Stimulation

hAD‐MSCs were seeded onto sterile stainless‐steel plates (3.8 × 2.8 × 0.7 cm) at a density of 2 × 10^4^ cells/cm^2^ and incubated for 24 h at 37°C with 5% CO_2_. The cells were randomly divided into control (unstimulated) and two ES groups. A two‐electrode system was employed, in which a platinum mesh (5 × 10 mm) and the cell‐seeded stainless‐steel plate served as the counter and working electrodes, respectively, using a multichannel potentiostat (VersaSTAT3; Princeton Applied Research, Oakridge, TN, USA). Stimulation was applied at 0.3 V, 5 ms pulse duration, and frequencies of 1 Hz (LF) or 100 Hz (HF) for 2 h, according to previous studies with some modifications [45, 46, 47, 48].

### 5.3 In Vitro Cytocompatibility Test

One day after ES, the viability and metabolic activity of MSCs were assessed. Cell viability measured using the LIVE/DEAD viability/cytotoxicity kit (Invitrogen, Carlsbad, CA, USA) according to the manufacturer's protocol. Fluorescence images were acquired using a fluorescence microscope (DMI3000 B; Leica). Cell viability was expressed as the percentage of live (green) cells among total cells. The metabolic activity was measured using WST‐1 assay (DoGen, Seoul, Republic of Korea). After 1 h of incubation in WST‐1 solution (1:10 in the media) at 37°C, the absorbance of the supernatant at 450 nm was measured using a microplate reader (Varioskan LUX; Thermo Fisher Scientific) and were normalized to the unstimulated group.

### 5.4 Cellular Morphology

Cells were fixed in 3.7% w/v paraformaldehyde at 25°C for 20 min, permeabilized/blocked (0.1% v/v Triton X‐100, 4% w/v BSA, and 2% v/v goat serum in Dulbecco's phosphate buffered saline [DPBS]; Gibco) at 25°C for 1 h, and stained with Alexa Fluor 488‐conjugate phalloidin solution (1:200; Thermo Scientific) at 25°C for 30 min in the dark. Nuclei were stained with DAPI (1:1000 in DPBS) at 25°C for 3 min. Fluorescence images were acquired using a fluorescence microscope. Cell area and aspect ratio were measured using ImageJ software by tracing cell boundaries. For each group, measurements were obtained from 20 to 40 cells selected from five randomly chosen fields per replicate (*n* = 4).

### 5.5 Quantitative RT‐PCR Analysis

After 24‐h culture in the growth medium, total RNA was extracted from unstimulated and stimulated cells using TRIzol (Thermo Scientific). RNA concentration and purity were assessed by UV–visible spectroscopy (BioDrop Duo; BioDrop, UK). Complementary DNA was synthesized using a High‐Capacity cDNA Reverse Transcription Kit (Applied Biosystems). qRT‐PCR was performed using Power SYBR Green PCR Master Mix and a StepOnePlus Real‐Time PCR system under standard cycling conditions: 95°C for 10 min, followed by 40 cycles of 95°C for 15 s and 60°C for 1 min. Expression levels of VEGF, HGF, bFGF, and IGF‐1 were normalized to glyceraldehyde‐3‐phosphate dehydrogenase (GAPDH) using the 2^−ΔΔCt^ method [49]. Primer sequences are listed in Table S1. All reactions were performed in triplicate, and results were presented as fold changes relative to the unstimulated control.

### 5.6 Collection of CM

Two hours after ES, cells were washed twice with DPBS and incubated in serum‐free MEM‐α for additional 72 h. CM were collected, centrifuged at 2000 rpm for 30 min at 4°C to remove debris, filtered using a 0.2 µm filter, and stored at −80°C for further analysis.

### 5.7 Enzyme‐Linked Immunosorbent Assay

Human VEGF‐A and human HGF in the CM were quantified using ELISA kits (Elabscience, Houston, TX, USA) according to the manufacturer's protocols. The absorbance at 450 nm was measured using a microplate reader.

### 5.8 Growth Factor Antibody Array

Growth factors in the CM were analyzed using a Human Growth Factor Antibody Array Kit (RayBiotech, GA, USA) according to the manufacturer's protocols. Antibody‐printed membranes were incubated in blocking buffer at 25°C for 30 min, incubated overnight with CM at 4°C, then sequentially incubated with biotinylated antibody cocktail for 2 h and HRP–streptavidin solution at 25°C for 2 h. Detection buffer was applied onto the membranes [50]. Signals were detected using a chemiluminescence imaging system (Gel doc; Bio‐Rad, ChemiDoc XRS) with Image Lab Software. Signal intensities were normalized to those of positive controls.

### 5.9 Quantitative RNA Sequencing and Data Analysis

Total RNA was extracted from 1 × 10^6^ cells and subjected to Quantitative RNA sequencing (Quant‐Seq) analysis (Ebiogen, Seoul, Republic of Korea). Raw read counts were normalized using the Trimmed Mean of *M*‐values method and transformed to count‐per‐million. The results were analyzed using ExDEGA software (Ebiogen). DEGs (fold‐changes ≥ 1.5, *p* ≤ 0.05) were visualized as a volcano plot. Pathway enrichment analysis was performed using the DAVID (http://david.abcc.ncifcrf.gov/) and QuickGO (https://www.ebi.ac.uk/QuickGO/) databases. Ninety‐nine genes were represented as a heatmap using the MultiExperiment Viewer software (J. Craig Venter Institute, Rockville, MD, USA, version 4.9.0). Ten key GO biological processes were identified. Protein–protein interactions among DEGs were analyzed using the STRING v12.0, web tool (http://string‐db.org/), with k‐means clustering (three clusters).

### 5.10 Proteomics Analysis

CM were concentrated using a Vivaspin Turbo 15 RC (3 kDa cutoff; Satorius, Göttingen, Germany). Protein concentration was measured using the Bradford assay. Proteins (30 µg) were reduced with 1,4‐dithiothreitol (Sigma–Aldrich, St. Louis, MO, USA), alkylated with iodoacetamide (Sigma–Aldrich), and digested overnight with trypsin according to the literature [51]. Samples were desalted using C18 spin columns (Thermo Scientific) and analyzed by liquid chromatography‐tandem mass spectrometry using an Ultimate 3000 RSLC system coupled with a Q‐Exactive Orbitrap Plus mass spectrometer (Thermo Scientific). Peptides were separated with a 0.1% formic acid gradient at 3 µL/min. Data were processed using Thermo Scientific Xcalibur software, and protein identification/quantification was performed using Proteome Discoverer (Thermo Fisher Scientific). Intensities were normalized using the total peptide amount method, log2‐transformed, and median‐centered. Pathway enrichment analysis was performed using WikiPathway gene sets in Enrichr‐KG (https://maayanlab.cloud/enrichr‐kg/, adjusted *p* < 0.05). Protein–protein interactions were analyzed using STRING v12.0. All analyses were performed in triplicate.

### 5.11 Scratch Closure Assay

HUVECs (5 × 10^4^ cells/well) were seeded in 24‐well plates and cultured until 90% confluence. A scratch was made using a 200 µL pipette tip, and detached cells were removed by DPBS washing. CM from MSCs or epMSCs was mixed with serum‐free ECM and added to the cell plates. A 1:1 mixture of serum‐free MEM‐α and serum‐free ECM was used as the control. Cells were incubated at 37°C with 5% CO_2_. Wound closure was periodically monitored via optical microscopy (DMI3000B; Leica) for up to 12 h.

### 5.12 Tube Formation Assay

HUVECs were cultured in a serum‐free medium for 12 h and seeded onto Matrigel (Corning, New York City, NY, USA)‐coated 24‐well plates (1.2 × 10^5^ cells/well). A 1:1 mix of serum‐free ECM with either serum‐free MEM‐α, MSC CM, or epMSC CM was added. Cells were incubated at 37°C with 5% CO_2_ for 8 h. Optical micrographs were acquired 4 h after seeding. ImageJ software (National Institute of Health, Bethesda, MD, USA) was used to analyze the number of branching points and loops.

### 5.13 In Vivo Matrigel Plug Assay

All animal experiments were approved by the Committee on Animal Research and Ethics of Gwangju Institute of Science and Technology (GIST, Republic of Korea (approval number: GIST‐2021‐086)). Six‐week‐old male BALB/c athymic nude mice were anesthetized using 2% isoflurane (Ankuk Inc., Republic of Korea). Matrigel mixed with either unsimulated MSCs or epMSCs (2 × 10^5^ cells/mL, 500 µL) was subcutaneously injected into both sides of the backs using an 18 G needle (*n* = 4 per group). One week after injection, the plugs were retrieved and analyzed by H&E staining, immunostaining for vWF, and hemoglobin quantification according to the literature [52].

### 5.14 HLI Model

Eight‐week‐old male BALB/c athymic nude mice were anesthetized using 2% isoflurane. Ischemia was induced through double‐knot ligation of the femoral and saphenous artery using 8‐0 silk sutures. MSCs (1.5 × 10^6^ cells in 50 µL DPBS) were injected into the adductor muscle (approximately 3 mm depth) using a 27‐gauge needle. The incision was closed using 6‐0 silk suture. Experimental groups included control (PBS), MSCs, and epMSCs.

### 5.15 Laser Doppler Perfusion Imaging and Limb Salvage Measurement

Hindlimb blood perfusion was assessed on days 0, 3, 7, and 14 after surgery using PeriScan PIM 3 (Perimed AB, Stockholm, Sweden). Mice were anesthetized via intraperitoneal injection of ketamine/xylazine mixture (4:1, 300 µL). Blood flow from the knee joint to the toe (2.5 × 3.5 cm) was measured from color‐coded digital images. On day 14, limb condition was graded as limb loss, foot necrosis, toe necrosis, and limb salvage (*n* = 6 per group).

### 5.16 Histological Analyses of Ischemic Limbs

On day 14, gastrocnemius and quadriceps of ischemic limbs were harvested, fixed, dehydrated, and paraffinized [53]. Tissue sections (5 µm) were stained with H&E and MT, imaged using a VS200 Research Slide Scanner (OLYMPUS, Tokyo, Japan). Fibrosis was quantified from the MT‐stained images as the percentage of the fibrotic (blue) area relative to the injured area using ImageJ. For immunofluorescence staining, the sections were incubated in the blocking solution (3% goat serum in DPBS) at 25°C for 1 h, incubated in the primary antibody solution (anti‐vWF (Chemicon, Temecula, CA, USA)) or anti‐HNA (Abcam, Cambridge, UK); 1:100 in 0.05% Triton‐X 100 and 1.5% goat serum) at 4°C overnight, washed twice with DPBS, and incubated in Alexa Fluor 555‐ or Alexa Fluor 488‐conjugated secondary antibody solution (1:200 in DPBS; Invitrogen) at 37°C for 1 h. Autofluorescence was quenched with 0.1% Sudan black B, and nuclei were stained with DAPI. Fluorescence images were acquired using a Research Slide Scanner. The vWF‐ or HNA‐positive areas were measured using ImageJ and normalized to the corresponding DAPI‐positive areas.

### 5.17 Statistical Evaluation

All tests were performed at least three times and results were reported as mean ± standard deviation unless otherwise noted. Data normality was assessed using the Shapiro–Wilk test. Group comparisons were performed using one‐way analysis of variance followed by Tukey's posthoc test for multiple comparisons in OriginPro software (OriginLab Corporation, Northampton, MA, USA). For transcriptomic and proteomic analyses, *p* values were adjusted for the false discovery rate method, in which a false discovery rate <0.05 was considered statistically significant. Additional statistical details are provided in each relevant subsection.

## Author Contributions

Jongdarm Yi: conceptualization, data curation, formal analysis, investigation, methodology, validation, visualization, writing—original draft. Seungjun Lee: data curation, formal analysis, investigation, methodology, validation, visualization, writing—review and editing, and writing—original draft. Chiseon Ryu: formal analysis, investigation, and validation. Gaeun Kim: methodology, investigation, and validation. Junghyun Kim: investigation and validation. Jae Young Lee: supervision, writing—review and editing, writing—original draft, conceptualization, and funding acquisition. All authors have read and approved the final manuscript.

## Ethics Statement

All experiments involving animals were approved by the Committee on Animal Research and Ethics of Gwangju Institute of Science and Technology (GIST, Republic of Korea (approval number: GIST‐2021‐086)).

## Conflicts of Interest

All authors declare no conflicts of interests.

## Supporting information




**Supporting Table 1**: List of primer sequences for real‐time qPCR.

**Supporting Fig 1**: (a) Schematic illustration and (b) real images of the lab‐established ES system. A SUS316L stainless steel plate and platinum mesh were used as a working electrode and a counter electrode, respectively.

**Supporting Fig 2**: (A) Fluorescence staining for F‐actin and nuclei of electrically non‐stimulated or stimulated MSCs on stainless steel plates. Scale bar = 200 µm. Quantitative analyses of (B) cell area and (C) aspect ratio between electrically stimulated and non‐stimulated MSCs. For each group, measurements were obtained from 20–40 cells selected from five randomly chosen fields per replicate (n = 4). An asterisk (*) denotes a statistically significant difference (p < 0.05).

**Supporting Fig 3**: (A) Quantification of VEGF and (B) HGF secretion in conditioned media from unstimulated MSCs and epMSCs at 12, 24, 48, and 72 hours post‐stimulation, measured by ELISA (n=4). An asterisk (*) denotes a statistically significant difference (p < 0.05) compared to the corresponding MSC control at each time point.

**Supporting Fig 4**: Growth factor secretion profiles of the MSCs and epMSCs. The rectangles highlight the expression of EGF, PDGF family, IGFBP family, HGF, and VEGF‐A.

**Supporting Fig 5**: In vivo Matrigel plug assay. (A) Representative images of the gross morphology, H&E, and vWF immunofluorescence of the plugs retrieved at 7 days after transplantation. Arrows indicate vessel‐like structures. Scale bars are 5mm, 100µm, and 200µm for morphology, H&E, and vWF/DAPI immunofluorescence images, respectively. (B) Hemoglobin contents in the plugs of the individual groups (n=4). Hemoglobin contents were normalized by the weights of harvested plugs. (C) vWF‐positive area, normalized by the 4',6‐diamidino‐2‐phenylindole (DAPI)‐positive areas in each group (n=4). An asterisk (*) denotes a statistically significant difference (*p* < 0.05).

**Supporting Fig 6**: STRING analysis of the protein networks identified 84 protein nodes among 99 DEGs. Ten proteins corresponding to the blood vessel development (GO:0001568) are highlighted in red.

**Supporting Fig 7**: Schematic representation of intramuscular MSC injection in the murine hindlimb ischemia (HLI) model. MSC were performed intramuscularly into the ischemic limb, specifically targeting the central region of the adductor muscle group. The injection was conducted approximately 3 mm deep into the muscle tissue to ensure accurate cell delivery and optimal engraftment.

**Supporting Fig 8**: Photographs of ischemic hindlimbs from all of the groups on Day 14 after transplantation (n=6).

**Supporting Fig 9**: Co‐localization analysis of HNA positive cells with endothelial marker (vWF) (n=5).

## Data Availability

The RNA‐seq data have been deposited in the NCBI Gene Expression Omnibus (GEO) under the accession number GSE300720 (https://www.ncbi.nlm.nih.gov/geo/query/acc.cgi?acc=GSE300720). The mass spectrometry proteomics data have been deposited in the ProteomeXchange Consortium via the PRIDE partner repository with the accession number PXD066035 [54]. All other data supporting the findings of this study are available from the corresponding author upon reasonable request.
