# Supplementary material for: Enhanced Angiogenic Potential of Electrically Stimulated Human Adipose‐Derived Mesenchymal Stem Cells (MSCs) for Ischemic Tissue Regeneration
Source: MedComm (2020). 2025 Sep 9;6(9):e70352. doi: 10.1002/mco2.70352 (PMC12421068; doi:10.1002/mco2.70352)
Supplement: Supplementary file 1 — Supporting Table 1: List of primer sequences for real‐time qPCR. [file MCO2-6-e70352-s004.docx]

**Enhanced Angiogenic Potential of Electrically Stimulated Human Adipose-Derived MSCs for Ischemic Tissue Regeneration**

Jongdarm Yi^1,#^, Seungjun Lee^1,#^, Chiseon Ryu^1^, Gaeun Kim^1^, Junghyun Kim^1^, and Jae Young Lee^1,*^

^1^ School of Materials Science and Engineering, Gwangju Institute of Science and Technology (GIST), Gwangju, 61005, Republic of Korea

* **Correspondence**

Jae Young Lee, *School of Materials Science and Engineering, Gwangju Institute of Science and Technology (GIST), Gwangju, 61005, Republic of Korea.*
Email: jaeyounglee@gist.ac.kr

**^#^**Jongdarm Yi, Seungjun Lee authors have contributed equally to this work.


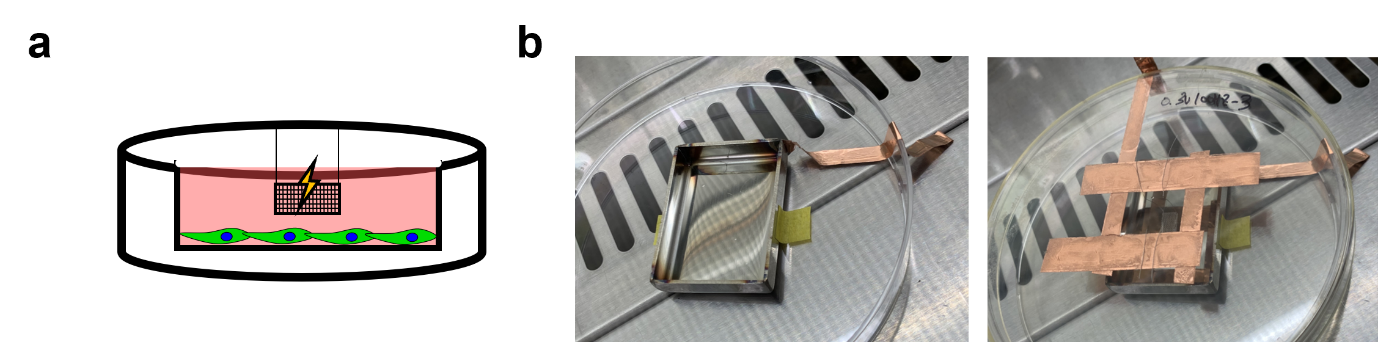


**Figure S1.** (a) Schematic illustration and (b) real images of the lab-established ES system. A SUS316L stainless steel plate and platinum mesh were used as a working electrode and a counter electrode, respectively.


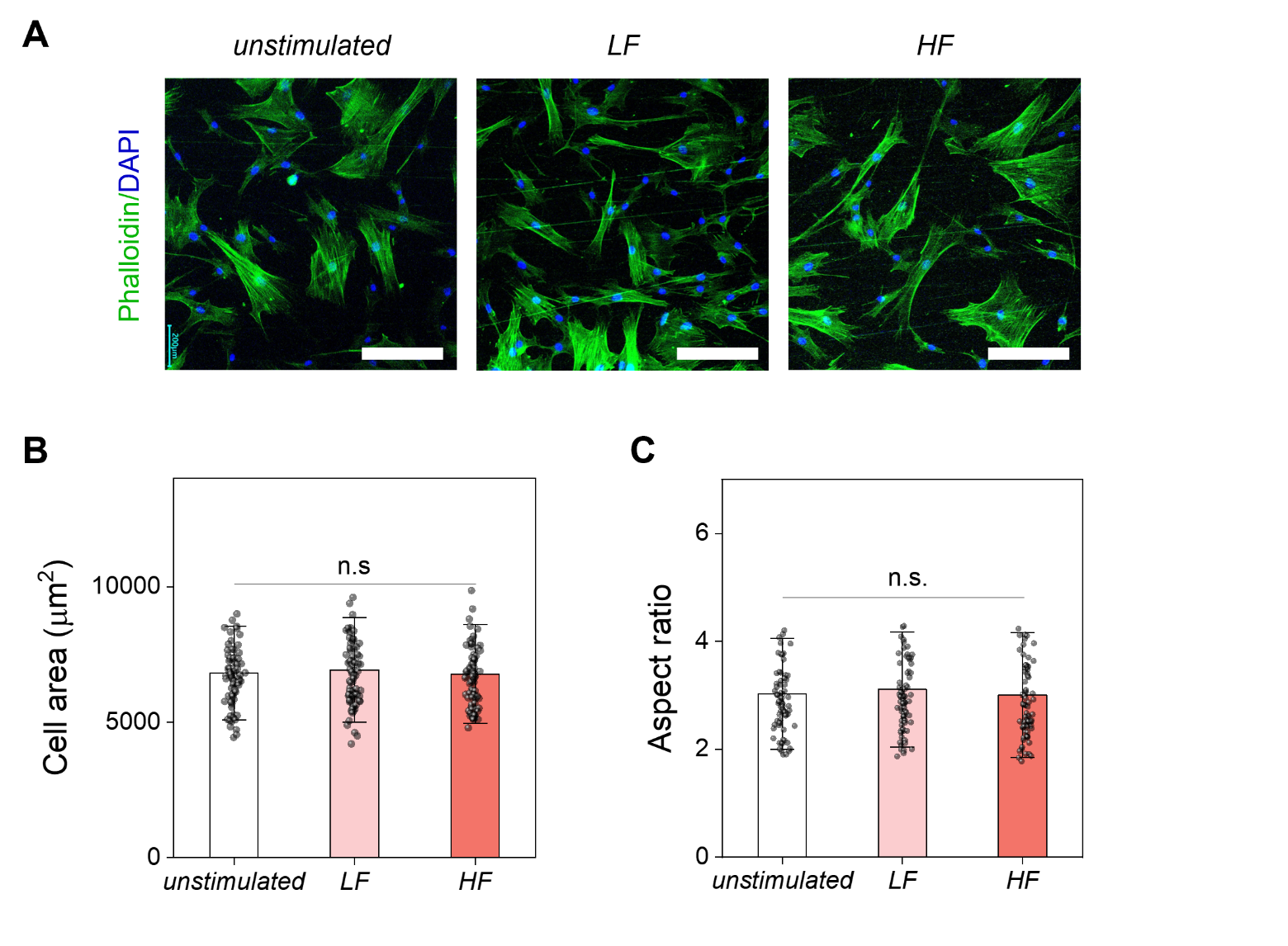
**Figure S2.** (A) Fluorescence staining for F-actin and nuclei of electrically non-stimulated or stimulated MSCs on stainless steel plates. Scale bar = 200 μm. Quantitative analyses of (B) cell area and (C) aspect ratio between electrically stimulated and non-stimulated MSCs. For each group, measurements were obtained from 20–40 cells selected from five randomly chosen fields per replicate (n = 4). An asterisk (*) denotes a statistically significant difference (p < 0.05).

**Figure S3.** (A) Quantification of VEGF and (B) HGF secretion in conditioned media from unstimulated MSCs and epMSCs at 12, 24, 48, and 72 hours post-stimulation, measured by ELISA (n=4). An asterisk (*) denotes a statistically significant difference (p < 0.05) compared to the corresponding MSC control at each time point.

**A**

**B**


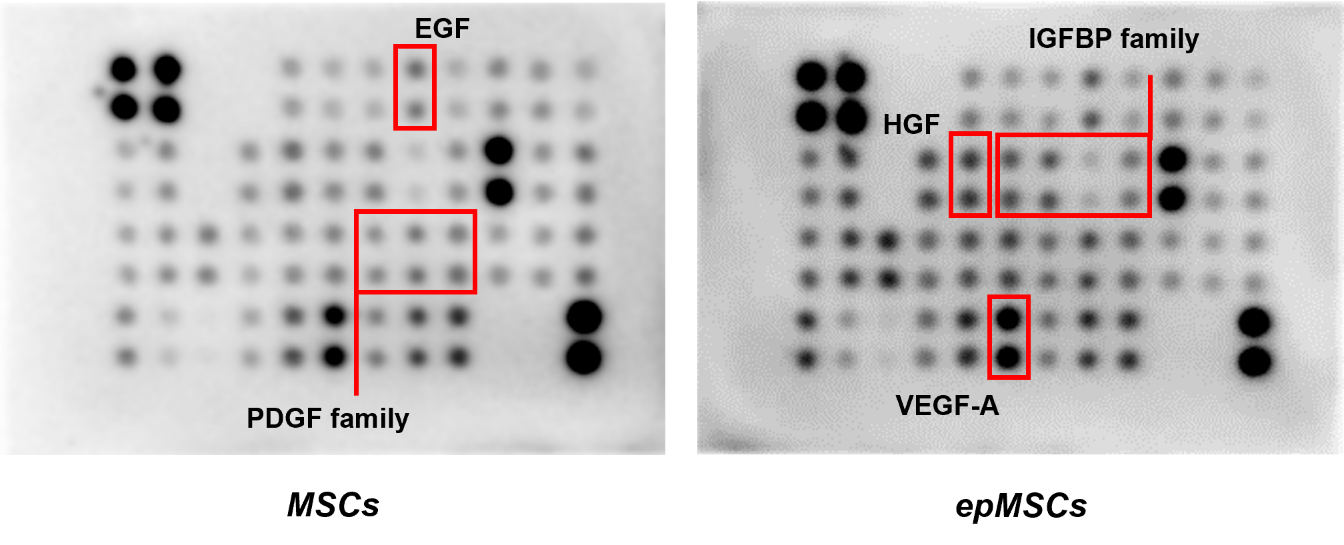


**Figure S4.** Growth factor secretion profiles of the MSCs and epMSCs. The rectangles highlight the expression of EGF, PDGF family, IGFBP family, HGF, and VEGF-A.

**
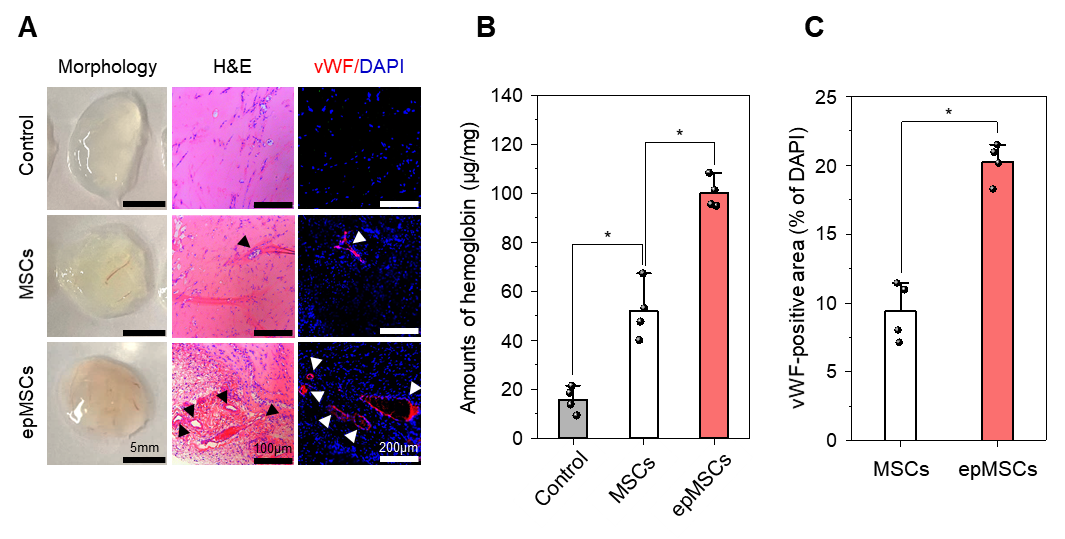
Figure S5.** *In vivo* Matrigel plug assay. (A) Representative images of the gross morphology, H&E, and vWF immunofluorescence of the plugs retrieved at 7 days after transplantation. Arrows indicate vessel-like structures. Scale bars are 5mm, 100μm, and 200μm for morphology, H&E, and vWF/DAPI immunofluorescence images, respectively. (B) Hemoglobin contents in the plugs of the individual groups (n=4) . Hemoglobin contents were normalized by the weights of harvested plugs. (C) vWF-positive area, normalized by the 4',6-diamidino-2-phenylindole (DAPI)-positive areas in each group (n=4). An asterisk (*) denotes a statistically significant difference (*p* < 0.05).


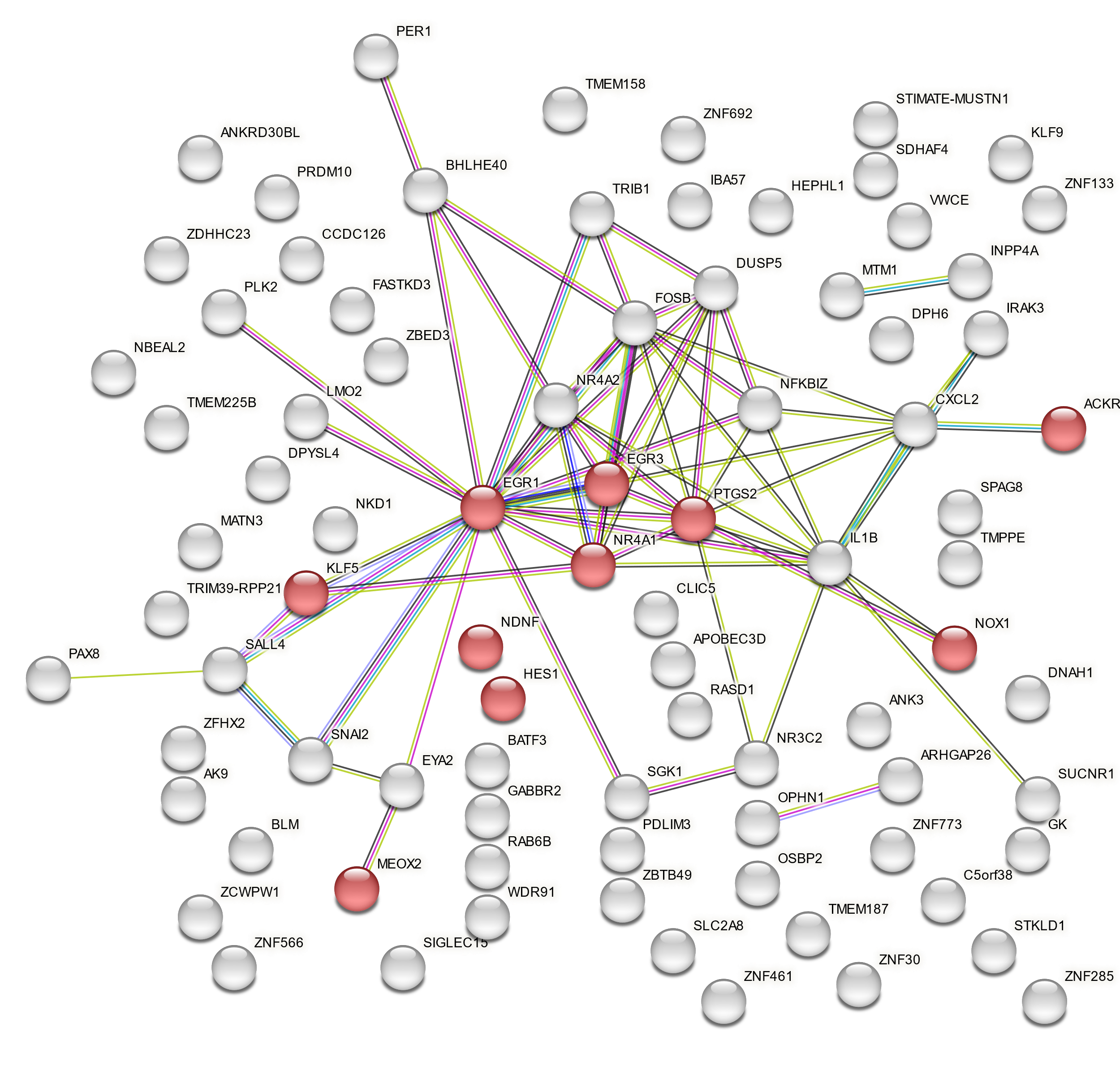


**Figure S6.** STRING analysis of the protein networks identified 84 protein nodes among 99 DEGs. Ten proteins corresponding to the blood vessel development (GO:0001568) are highlighted in red.


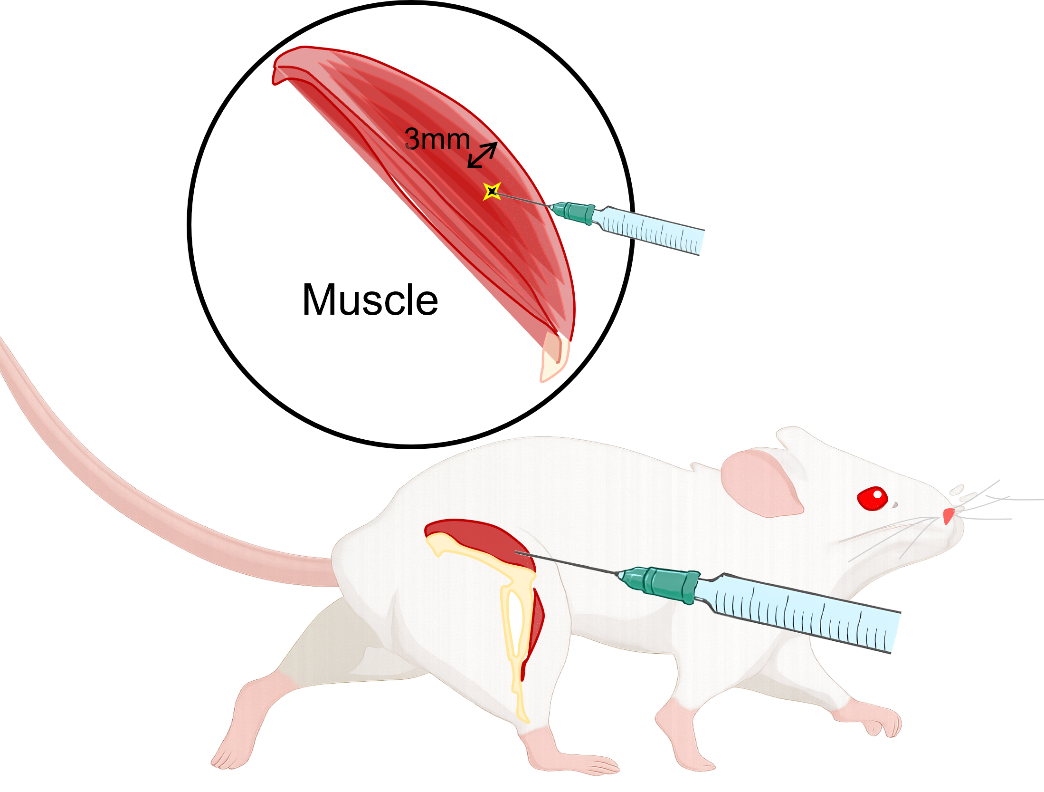
 **Figure S7**. Schematic representation of intramuscular MSC injection in the murine hindlimb ischemia (HLI) model. MSC were performed intramuscularly into the ischemic limb, specifically targeting the central region of the adductor muscle group. The injection was conducted approximately 3 mm deep into the muscle tissue to ensure accurate cell delivery and optimal engraftment.


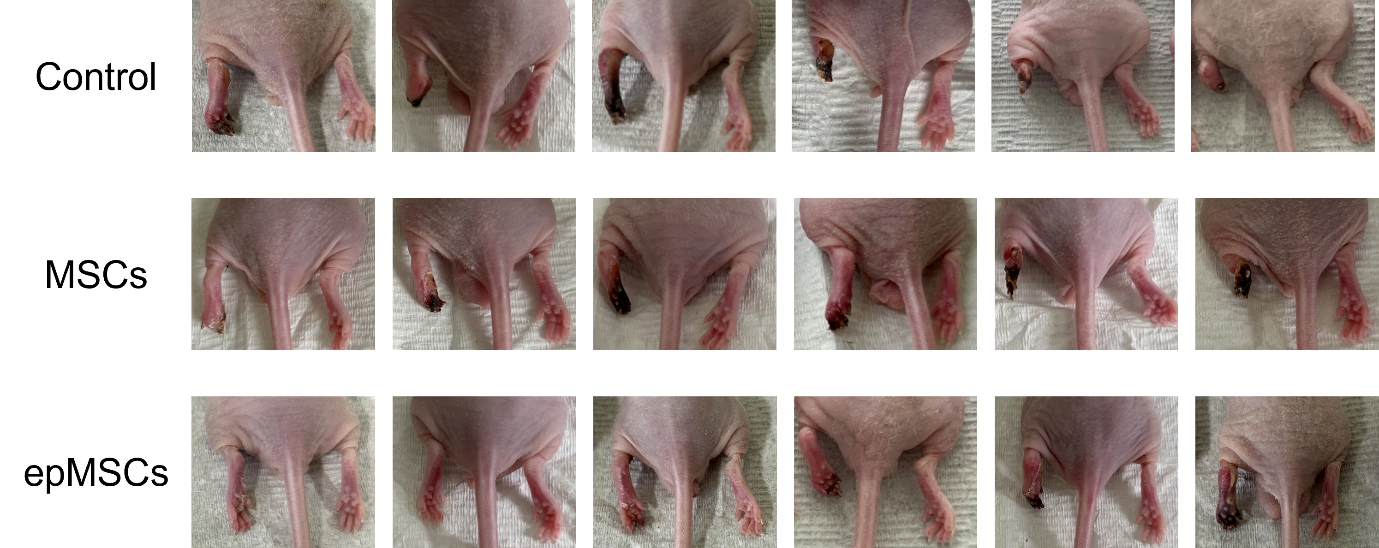


**Figure S8.** Photographs of ischemic hindlimbs from all of the groups on Day 14 after transplantation (n=6).

**
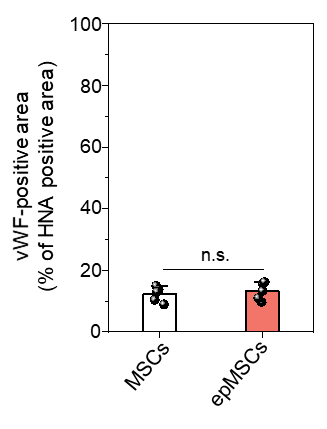
**

**Figure S9.** Co-localization analysis of HNA positive cells with endothelial marker (vWF) (n=5).

**Table S1.** List of primer sequences for real-time qPCR

| **Target gene** | **Forward** | **Reverse** |
| --- | --- | --- |
| *GAPDH* | ATT TGG TCG TAT TGG GCG | TGG AAG ATG GTG ATG GGA TT |
| *VEGF* | TCT TCA AGC CAT CCT GTG TG | ATC TGC ATG GTG ATG TTG GA |
| *HGF* | TGC TGT CCT GGA TGA TTT TG | AGT GTA GCC CCA GCC ATA AA |
| *bFGF* | GCT TCT TCC TGC GCA TCC A | CAG CTC TTA GCA GAC ATT GG |
| *IGF-1* | TCT GCA CGA GTT ACC TGT TA | CAA TCT ACC AAC TCC AGG AC |
