# Supplementary figures and images for: Enhanced Angiogenic Potential of Electrically Stimulated Human Adipose‐Derived Mesenchymal Stem Cells (MSCs) for Ischemic Tissue Regeneration
Source: MedComm (2020). 2025 Sep 9;6(9):e70352. doi: 10.1002/mco2.70352 (PMC12421068; doi:10.1002/mco2.70352)

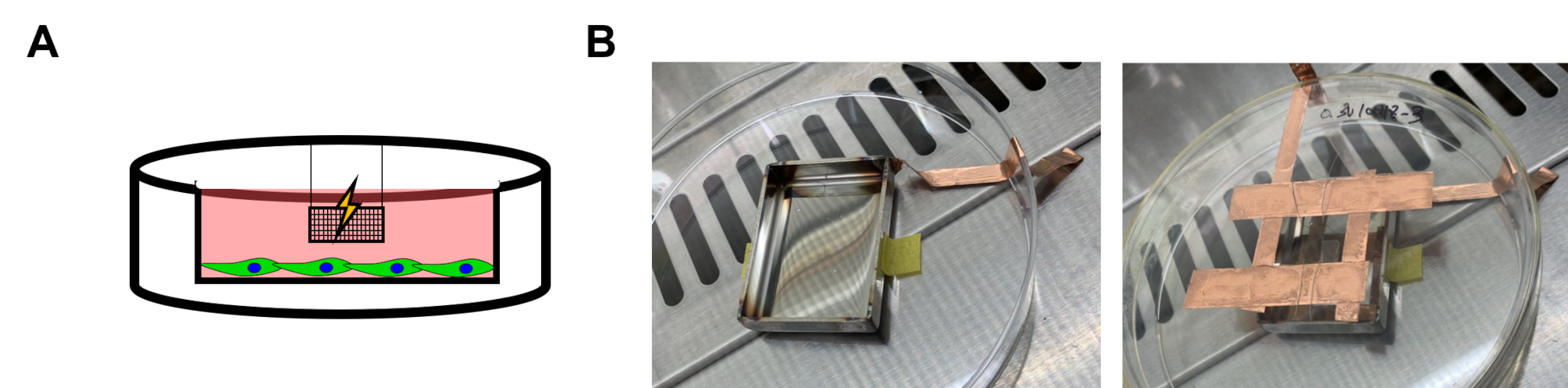

Supplement: Supplementary file 2 — Supporting Fig 1: (a) Schematic illustration and (b) real images of the lab‐established ES system. A SUS316L stainless steel plate and platinum mesh were used as a working electrode and a counter electrode, respectively. [file MCO2-6-e70352-s003.tif]

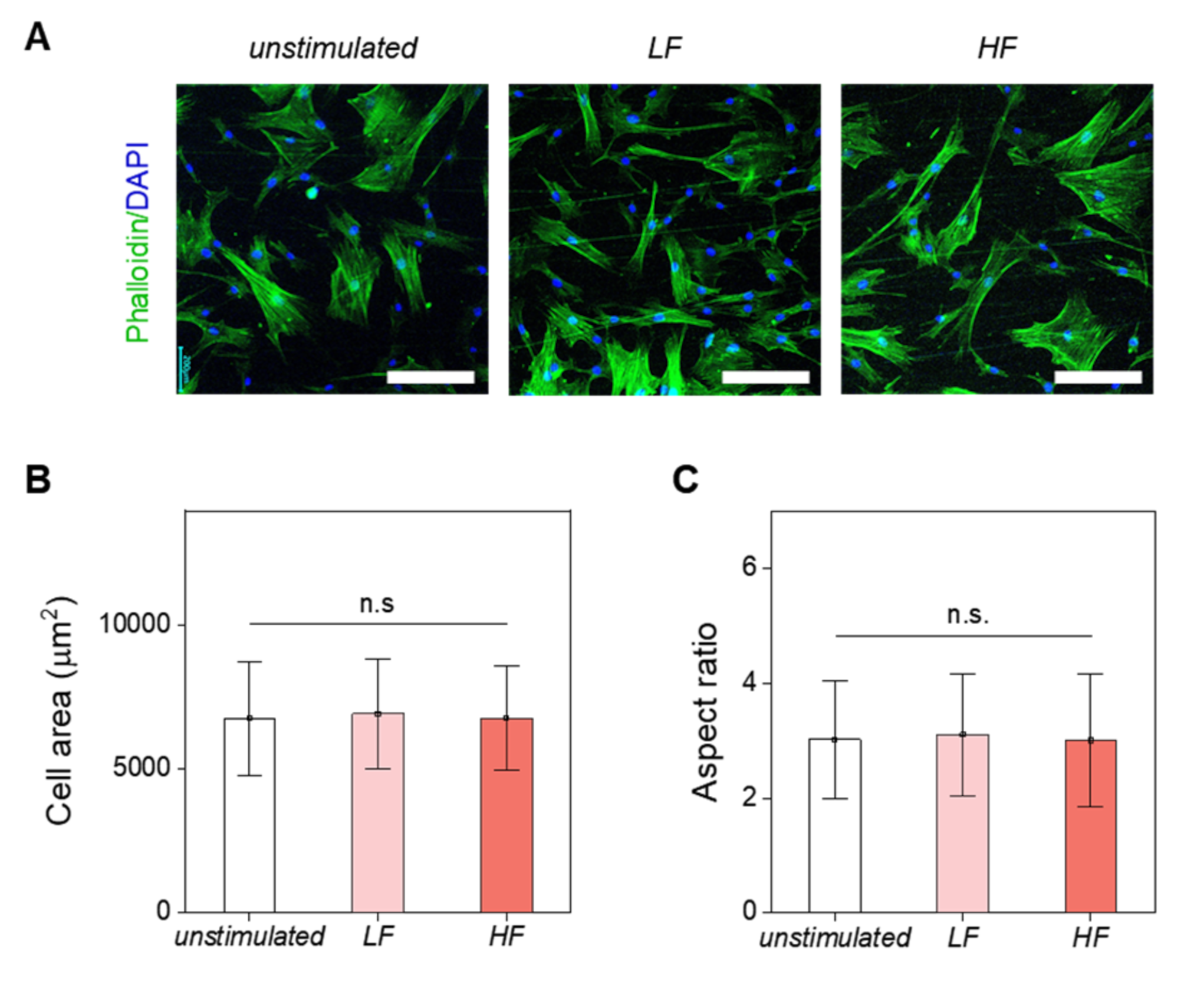

Supplement: Supplementary file 3 — Supporting Fig 2: (A) Fluorescence staining for F‐actin and nuclei of electrically non‐stimulated or stimulated MSCs on stainless steel plates. Scale bar = 200 µm. Quantitative analyses of (B) cell area and (C) aspect ratio between electrically stimulated and non‐stimulated MSCs. For each group, measurements were obtained from 20–40 cells selected from five randomly chosen fields per replicate (n = 4). An asterisk (*) denotes a statistically significant difference (p < 0.05). [file MCO2-6-e70352-s005.tif]

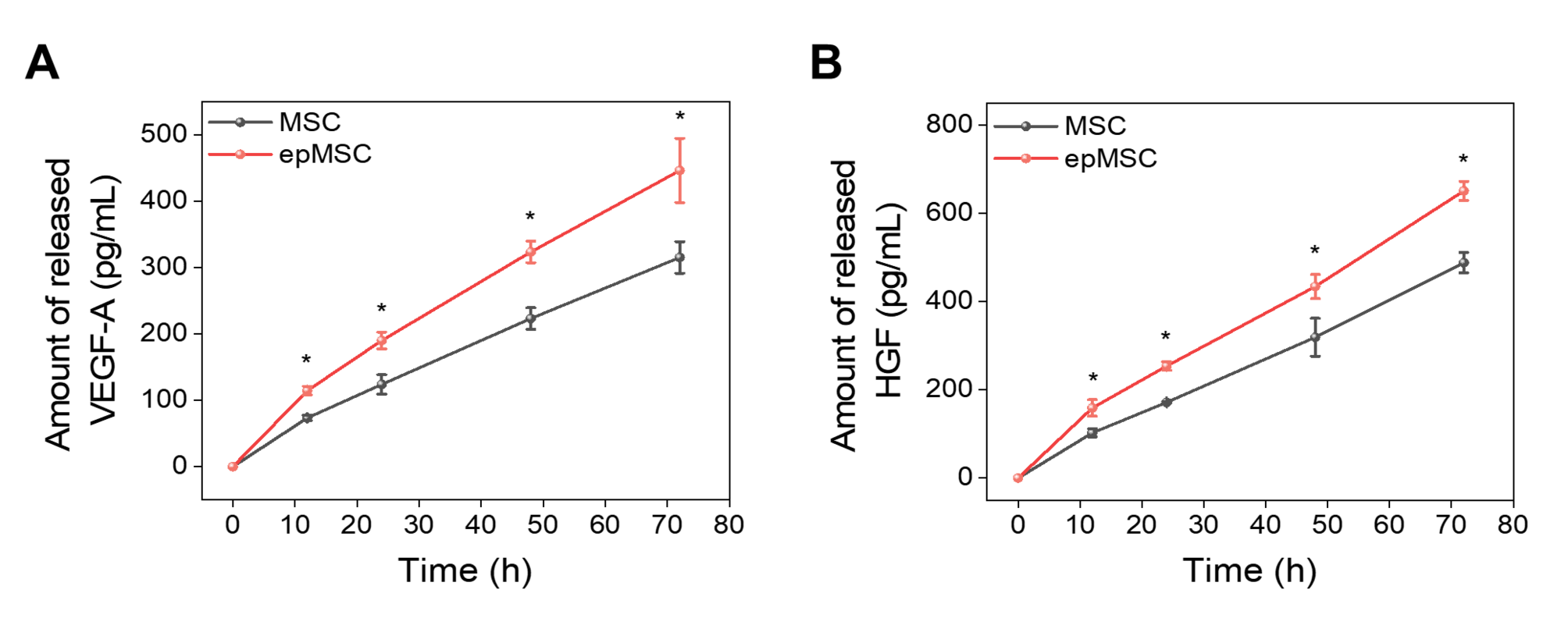

Supplement: Supplementary file 4 — Supporting Fig 3: (A) Quantification of VEGF and (B) HGF secretion in conditioned media from unstimulated MSCs and epMSCs at 12, 24, 48, and 72 hours post‐stimulation, measured by ELISA (n=4). An asterisk (*) denotes a statistically significant difference (p < 0.05) compared to the corresponding MSC control at each time point. [file MCO2-6-e70352-s007.tif]

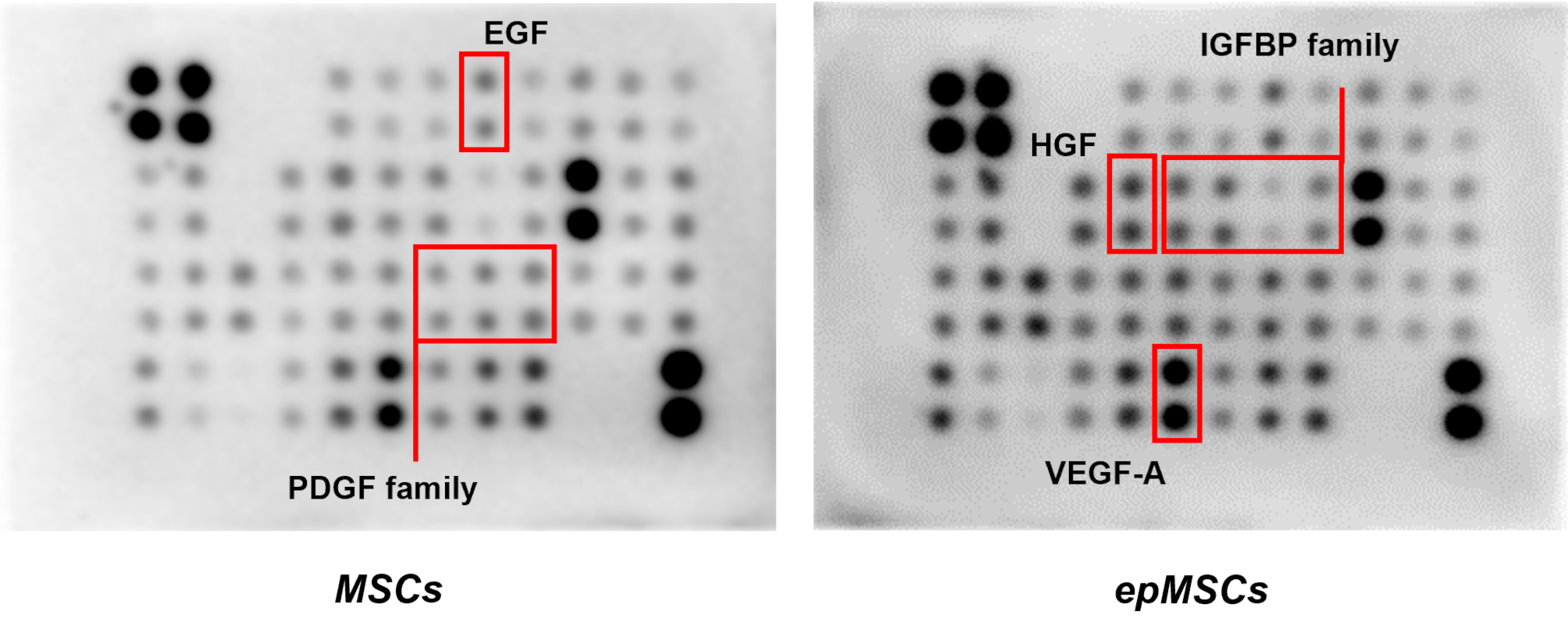

Supplement: Supplementary file 5 — Supporting Fig 4: Growth factor secretion profiles of the MSCs and epMSCs. The rectangles highlight the expression of EGF, PDGF family, IGFBP family, HGF, and VEGF‐A. [file MCO2-6-e70352-s006.tif]

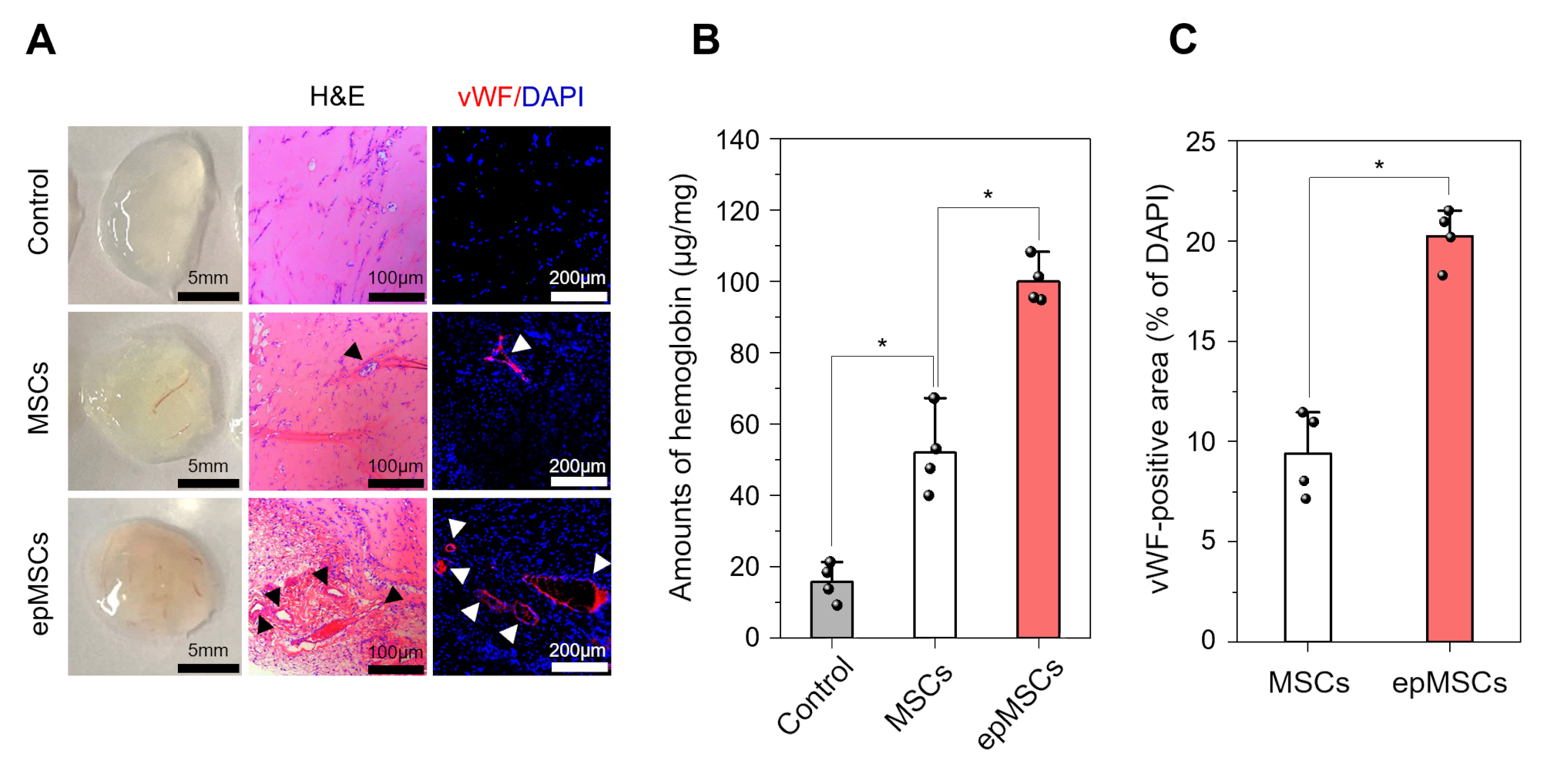

Supplement: Supplementary file 6 — Supporting Fig 5: In vivo Matrigel plug assay. (A) Representative images of the gross morphology, H&E, and vWF immunofluorescence of the plugs retrieved at 7 days after transplantation. Arrows indicate vessel‐like structures. Scale bars are 5mm, 100µm, and 200µm for morphology, H&E, and vWF/DAPI immunofluorescence images, respectively. (B) Hemoglobin contents in the plugs of the individual groups (n=4). Hemoglobin contents were normalized by the weights of harvested plugs. (C) vWF‐positive area, normalized by the 4',6‐diamidino‐2‐phenylindole (DAPI)‐positive areas in each group (n=4). An asterisk (*) denotes a statistically significant difference (p < 0.05). [file MCO2-6-e70352-s010.tif]

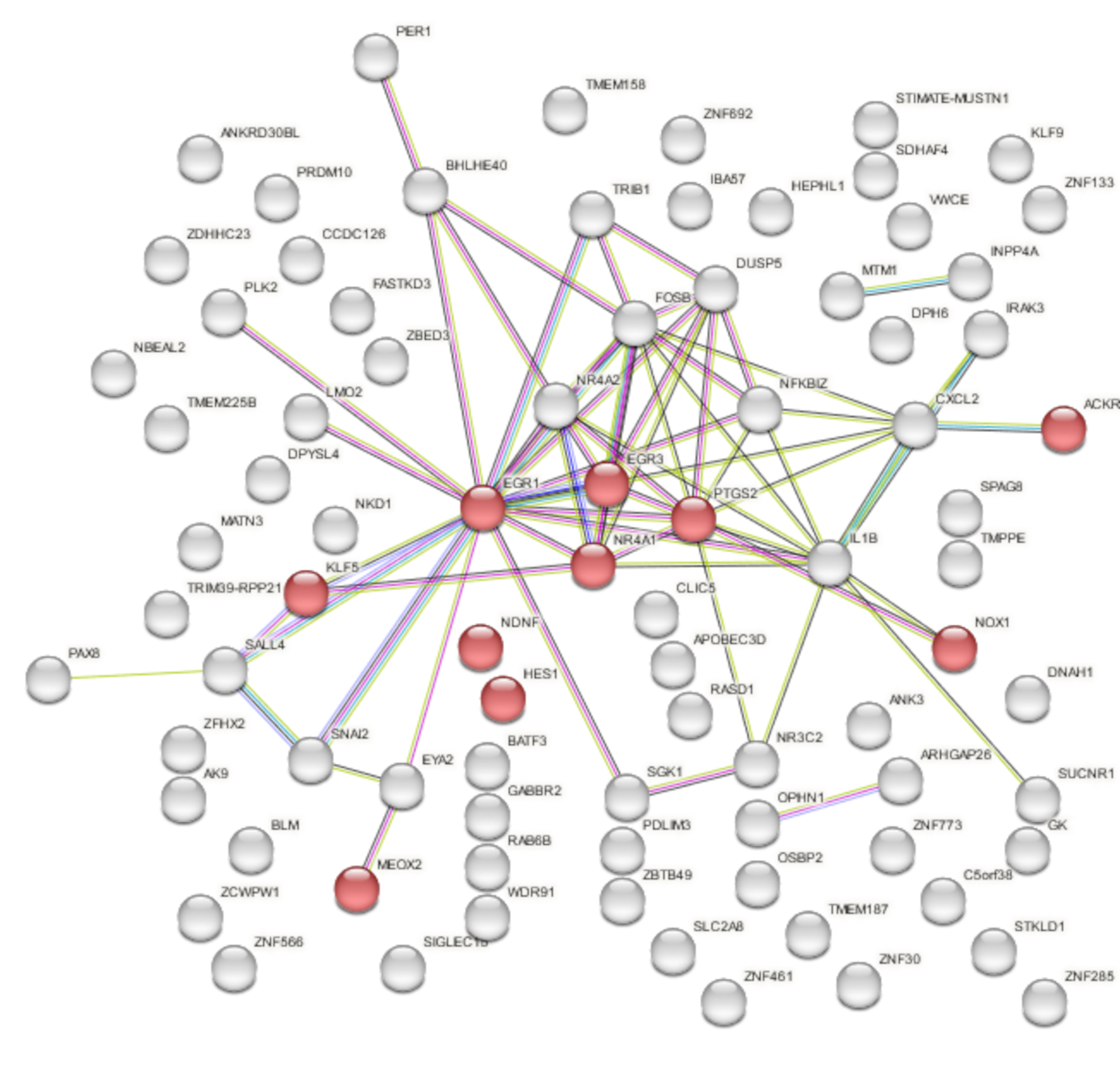

Supplement: Supplementary file 7 — Supporting Fig 6: STRING analysis of the protein networks identified 84 protein nodes among 99 DEGs. Ten proteins corresponding to the blood vessel development (GO:0001568) are highlighted in red. [file MCO2-6-e70352-s002.tif]

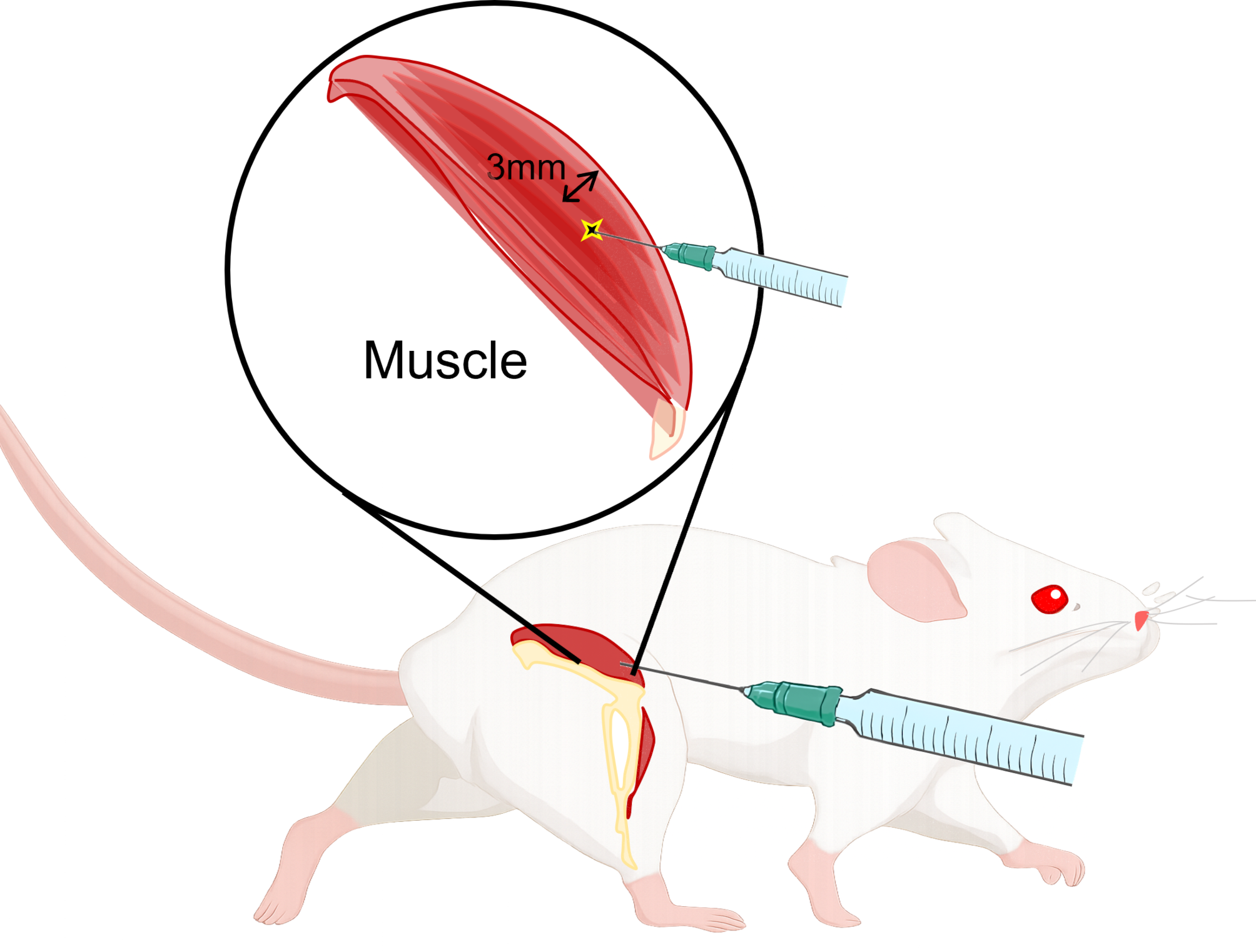

Supplement: Supplementary file 8 — Supporting Fig 7: Schematic representation of intramuscular MSC injection in the murine hindlimb ischemia (HLI) model. MSC were performed intramuscularly into the ischemic limb, specifically targeting the central region of the adductor muscle group. The injection was conducted approximately 3 mm deep into the muscle tissue to ensure accurate cell delivery and optimal engraftment. [file MCO2-6-e70352-s008.tif]

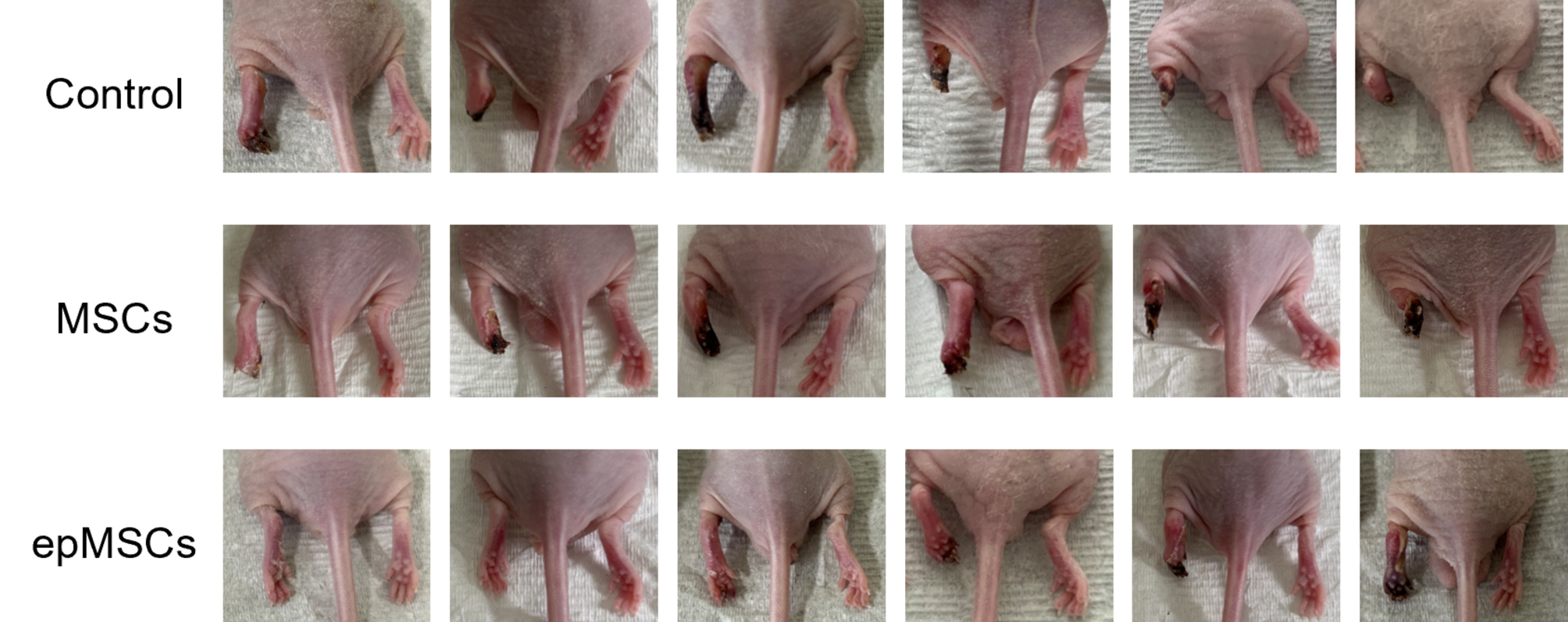

Supplement: Supplementary file 9 — Supporting Fig 8: Photographs of ischemic hindlimbs from all of the groups on Day 14 after transplantation (n=6). [file MCO2-6-e70352-s001.tif]

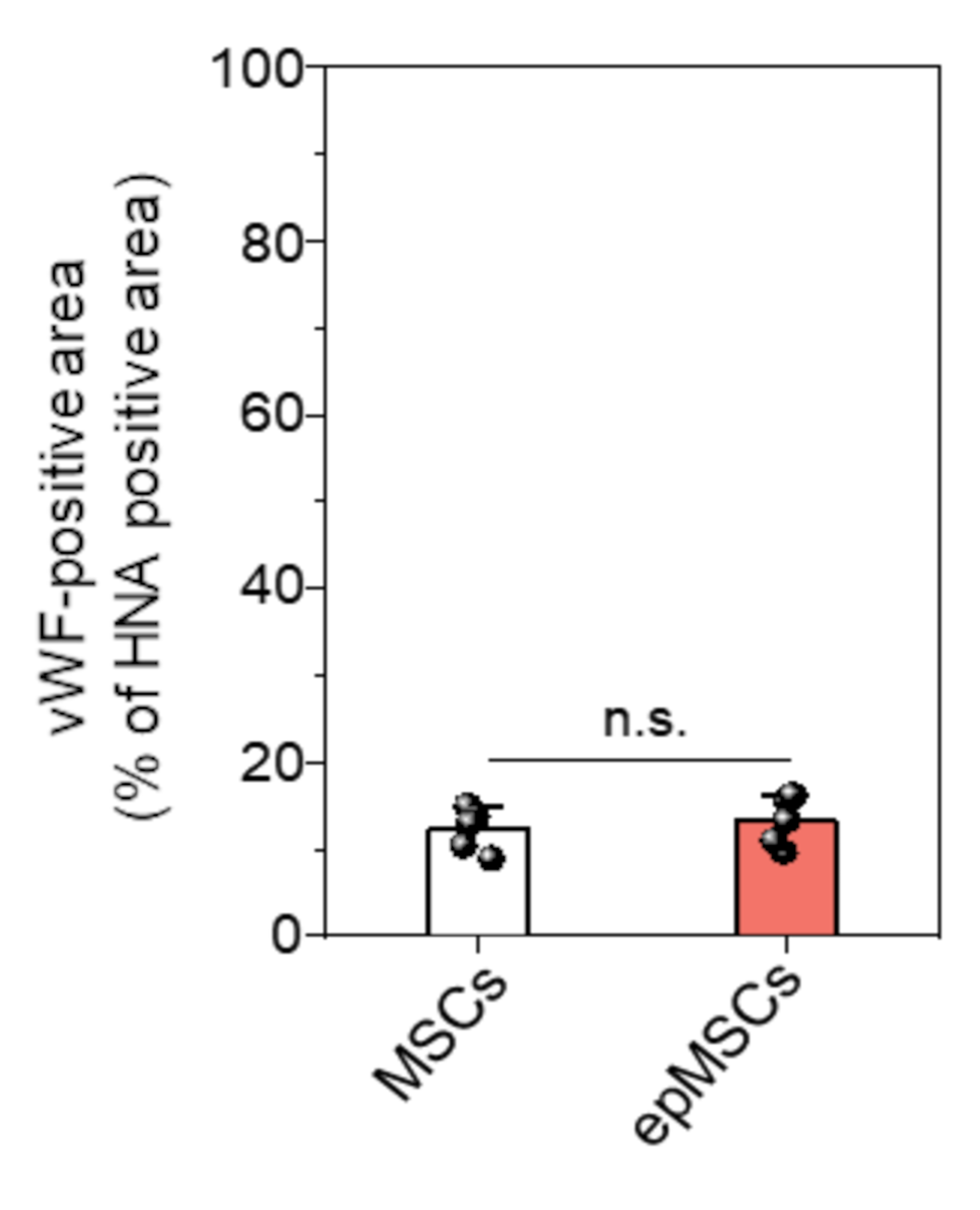

Supplement: Supplementary file 10 — Supporting Fig 9: Co‐localization analysis of HNA positive cells with endothelial marker (vWF) (n=5). [file MCO2-6-e70352-s009.tif]
